# Tissue specific human fibroblast differential expression based on RNAsequencing analysis

**DOI:** 10.1186/s12864-019-5682-5

**Published:** 2019-04-23

**Authors:** Alexander G. Foote, Ziyue Wang, Christina Kendziorski, Susan L. Thibeault

**Affiliations:** 1 Department of Surgery, Division of Otolaryngology – Head and Neck Surgery, University of Wisconsin, Madison, WI USA; 2 Department of Statistics, University of Wisconsin – Madison, College of Letters and Science, Madison, WI USA; 3 Department of Biostatistics & Medical Informatics, University of Wisconsin – Madison, Madison, WI USA

**Keywords:** Vocal fold biology, RNA-seq, Human vocal fold fibroblast, Functional gene expression, Transcriptome profiling, Mechanobiology, Mechanical force

## Abstract

**Background:**

Physical forces, such as mechanical stress, are essential for tissue homeostasis and influence gene expression of cells. In particular, the fibroblast has demonstrated sensitivity to extracellular matrices with assumed adaptation upon various mechanical loads. The purpose of this study was to compare the vocal fold fibroblast genotype, known for its unique mechanically stressful tissue environment, with cellular counterparts at various other anatomic locales to identify differences in functional gene expression profiles.

**Results:**

By using RNA-seq technology, we identified differentially expressed gene programs (DEseq2) among seven normal human fibroblast primary cell lines from healthy cadavers, which included: vocal fold, trachea, lung, abdomen, scalp, upper gingiva, and soft palate. Unsupervised gene expression analysis yielded 6216 genes differentially expressed across all anatomic sites. Hierarchical cluster analysis revealed grouping based on anatomic site origin rather than donor, suggesting global fibroblast phenotype heterogeneity. Sex and age-related effects were negligible. Functional enrichment analyses based on separate post-hoc 2-group comparisons revealed several functional themes within the vocal fold fibroblast related to transcription factors for signaling pathways regulating pluripotency of stem cells and extracellular matrix components such as cell signaling, migration, proliferation, and differentiation potential.

**Conclusions:**

Human fibroblasts display a phenomenon of global topographic differentiation, which is maintained in isolation via in vitro assays. Epigenetic mechanical influences on vocal fold tissue may play a role in uniquely modelling and maintaining the local environmental cellular niche during homeostasis with vocal fold fibroblasts distinctly specialized related to their anatomic positional and developmental origins established during embryogenesis.

**Electronic supplementary material:**

The online version of this article (10.1186/s12864-019-5682-5) contains supplementary material, which is available to authorized users.

## Background

Different tissue-types of the human body withstand and undergo various mechanical loads; encompassing either tension, compression, fluid shear, and/or torsional shear stresses [1]. These mechanical forces influence the differentiation state of cells, specifically the fibroblast, which demonstrate sensitivity to the surrounding extracellular matrix (ECM) [2–4]. The most abundant cell in the ECM, fibroblasts are a heterologous cell type that contribute to normal physiologic or pathologic conditions through either balance or imbalance between protein synthesis and degradation [5].

Recent studies have elucidated organ-dependent transcriptional diversity of human fibroblasts relative to anatomical position within and across the human body [6–8]. Results from prior work exploring anatomic demarcation by positional variation in fibroblasts suggest that site-specific variations in fibroblast gene expression are not individual or random, but rather, systematically related to their positional identities relative to three major anatomic axes: anterior-posterior, proximal-distal, and dermal versus nondermal [6]. Another study utilizing comprehensive gene expression analyses to examine diversity of human fibroblasts across the body revealed diverse transcriptional phenotypes based on organ- and site-specific anatomic location expressed by transcriptional regulation, humoral signaling ligands, and ECM remodeling [7]. In addition to distinct cell lineages based on anatomic site, it was found that adult fibroblasts maintained key features of *HOX* gene expression patterns established during embryogenesis. This suggests that topographic differentiation and positional memory are retained from embryonic development and underlie the key features maintained in adult fibroblasts [6, 8].

It is becoming increasingly apparent that physical forces are essential for tissue homeostasis and influence the gene expression of cells [9–13]. Many disease states associated with fibroblast functioning are characterized by diminished or excess deposition of the ECM, leading to changes in gene expression and tissue morphology [14, 15]. Previous work on human dermal fibroblasts established diversity of mechanotransduction properties and biochemical reactions in response to applied mechanical stress [16, 17]. As such, the responses of fibroblasts are assumed to be unique and adaptive, resulting in optimal modification and maintenance of their respective microenvironments. In this study, we sought to investigate differences in gene expression of fibroblasts that undergo mechanical loads for elucidation of genetic differences that may be advantageous to the surrounding ECM. Specifically, we utilized the vocal fold fibroblast (VFF) as an ideal surrogate cell-type for comparison due to its particular tissue environment that withstands high, chronic mechanical loading forces.

Human vocal folds (VFs) are a unique organ of the body; tissues are exposed to high inertial stresses [10]. Vibrations naturally occur at regular frequencies of 100–1000 Hz and amplitudes of about 1 mm [18]. Daily exposure times can vary between 1 and 2 h, with tissue accelerations reaching 200–300 G [19]. At present, many physiological and pathophysiological aspects of VFF function remain poorly understood, however it has been assumed that fibroblast function play a vital role in tissue function, normal tissue morphology, and mechanical support for tissues [20]. It is also thought that variations in homeostatic properties of the ECM contribute to pathogenesis of the underlying lamina propria (LP), including lesions, scarring, and sulcus vocalis [5].

Initial investigations have led to an increased understanding of the distinct and characteristic gene expression patterns of fibroblasts across anatomic sites, however there remains a gap in the literature with regard to incorporating non-dermal correlates, and more specifically, fibroblasts retained from highly mechanical tissue environments. The current study aims to characterize genome-wide patterns of gene expression in VFFs to determine whether they are distinctly differentiated cell types compared to other anatomic sites that lack high mechanical forces. Specific aims will utilize genome-wide expression profiling focused on investigating the transcriptional regulation, humoral signaling ligands, and ECM remodeling differences of VFFs to other fibroblast genotypes. Given the maturated microenvironment of VF tissue to support complex and unique mechanical forces, we hypothesize that resident fibroblasts have a globally specialized, diverse transcriptional genotype specific to their anatomic origins. We speculate, given prior literature on gene expression variations due to mechanical forces [2, 3, 9, 11, 12, 16, 17], that VFFs are particularly specialized compared to other anatomic sites that remain absent of such mechanical trauma.

## Results

**Fig. 1:**
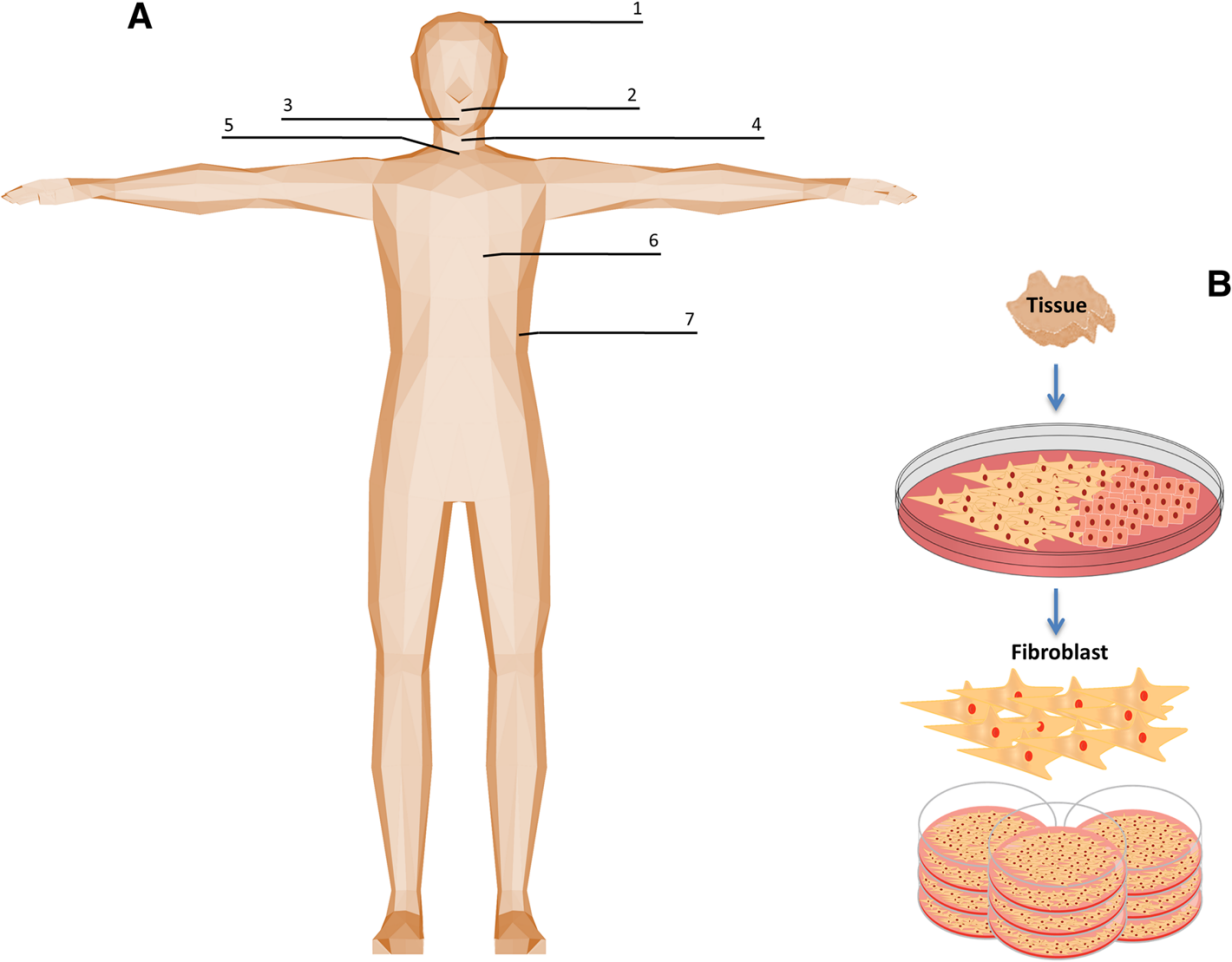
Experimental design for tissue procurement and fibroblast isolation. **a** Primary fibroblast populations, were obtained from 7 anatomic sites; scalp dermis (1), soft palate (2), upper gingiva (3), vocal fold (4), trachea (5), lung (6), and abdomen dermis (7). **b** Tissue explant methodology for heterogeneous cell populations with subsequent subcultures for isolation and purification of fibroblast colonies. Clipart was acquired and modified from clker.com and kisscc0.com and is part of Public Domain as stated under the CC0 1.0 license

**Fig. 2:**
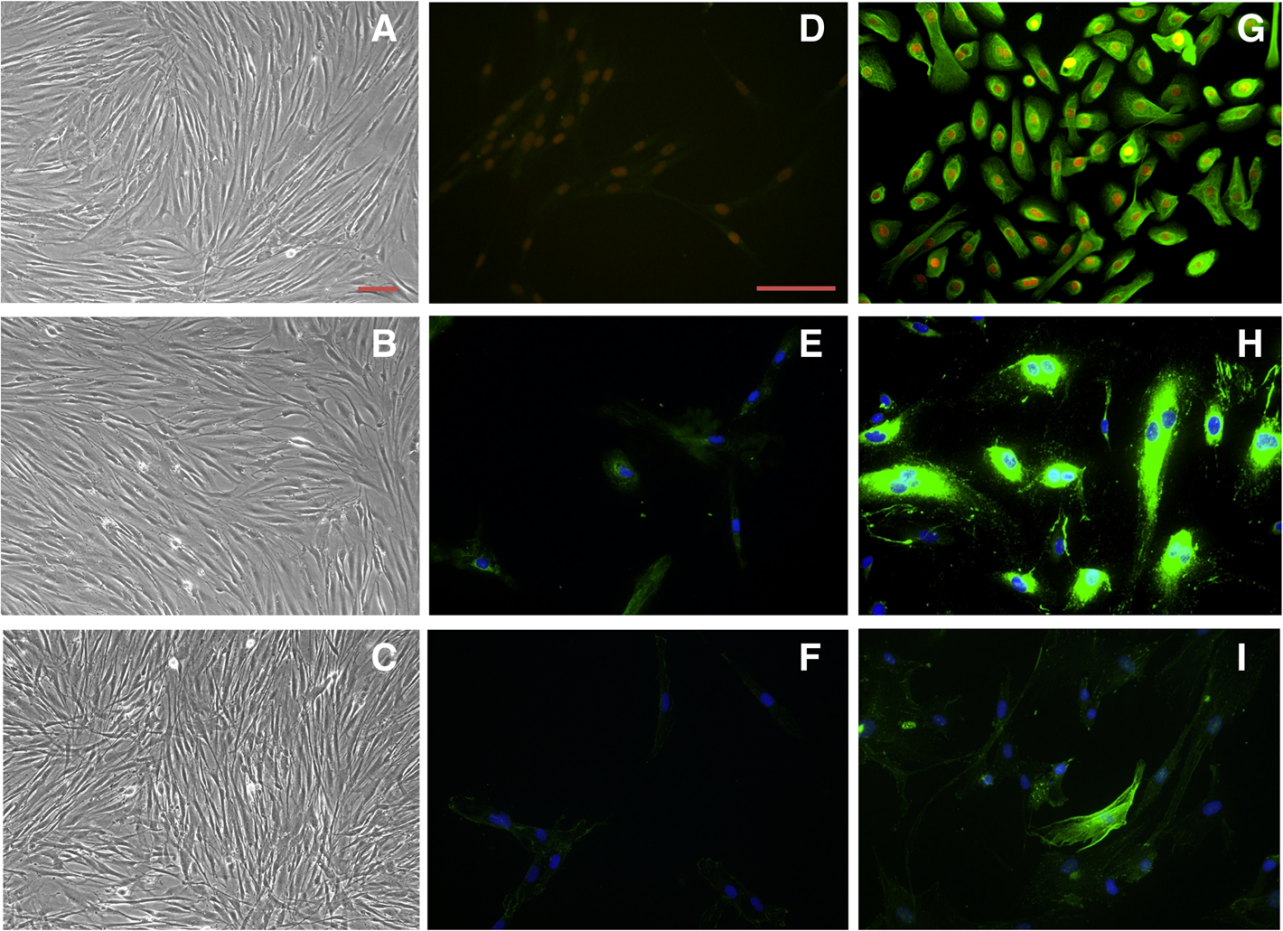
Lineage characterization of fibroblast cultures. Brightlight microscopy of vocal fold (**a)**, trachea (**b**), and lung (**c**) fibroblast cultures exhibiting homogenous populations with classic elongated, spindle-shape morphology. VFF negative for markers of cytokeratin 19 (**d**), mounted with propidium iodine, von Willebrand (vWF) (**e**), and α-actinin (**f**), mounted with DAPI. Epithelial cells exhibit positive (**g**) expression for cytokeratin 19. Endothelial cells exhibit positive (**h**) expression for vWF. Skeletal cells exhibit positive (**i**) expression for α-actinin. Experiments were performed in triplicates and with negative controls, both in the absence of primary antibodies and with known negative cell types. Brightfield photos taken at 10x and immunofluorescence photos taken at 20x magnification. Scale bar represents 100 μm

Thirty-three primary human fibroblast cultures were propagated in vitro*;* obtained from 7 different anatomic locales across 15 cadaveric donors with acquisition of 5 biological replicates from each anatomic location, with the exclusion of gingiva and palatal samples, in which each totaled 4 replicates, respectively (Fig. 1). Demographic information of successfully cultured fibroblasts from postmortem human tissue are summarized in Table 1. All cultured fibroblasts displayed similar elongated, spindle-shaped morphology regardless of anatomic derivative. Fibroblast lineage confirmation was performed by subtractive methodology [5], negative for markers of epithelial, endothelial, and skeletal muscle cells (Fig. 2).

**Table 1:** Demographic characteristics of successfully cultured fibroblasts from postmortem human tissue

Subject #	Site	Sex	Age
S01	Abdomen	M	74
S02	Lung	F	70
S03	Vocal fold	M	25
S03	Trachea	M	25
S03	Soft palate	M	25
S03	Scalp	M	25
S03	Upper Gingiva	M	25
S04	Soft Palate	M	75
S04	Vocal fold	M	75
S04	Upper Gingiva	M	75
S05	Soft Palate	F	32
S05	Upper Gingiva	F	32
S05	Abdomen	F	32
S05	Trachea	F	32
S05	Scalp	F	32
S06	Scalp	M	73
S06	Abdomen	M	73
S07	Trachea	F	32
S08	Vocal fold	M	89
S08	Trachea	M	89
S08	Upper Gingiva	M	89
S08	Lung	M	89
S09	Vocal fold	M	56
S09	Trachea	M	56
S09	Abdomen	M	56
S09	Scalp	M	56
S10	Abdomen	F	69
S10	Scalp	F	69
S11	Lung	M	61
S12	Vocal fold	F	58
S13	Soft Palate	F	69
S14	Lung	M	64
S15	Lung	F	37

Thirty-three primary human fibroblast cultures were propagated in vitro; obtained from 7 different anatomic locales across 15 cadaveric donors with acquisition of 5 biological replicates from each anatomic location, with the exclusion of gingiva and palatal samples, in which each totaled 4 replicates, respectively

### Unsupervised differential expression

**Fig. 3:**
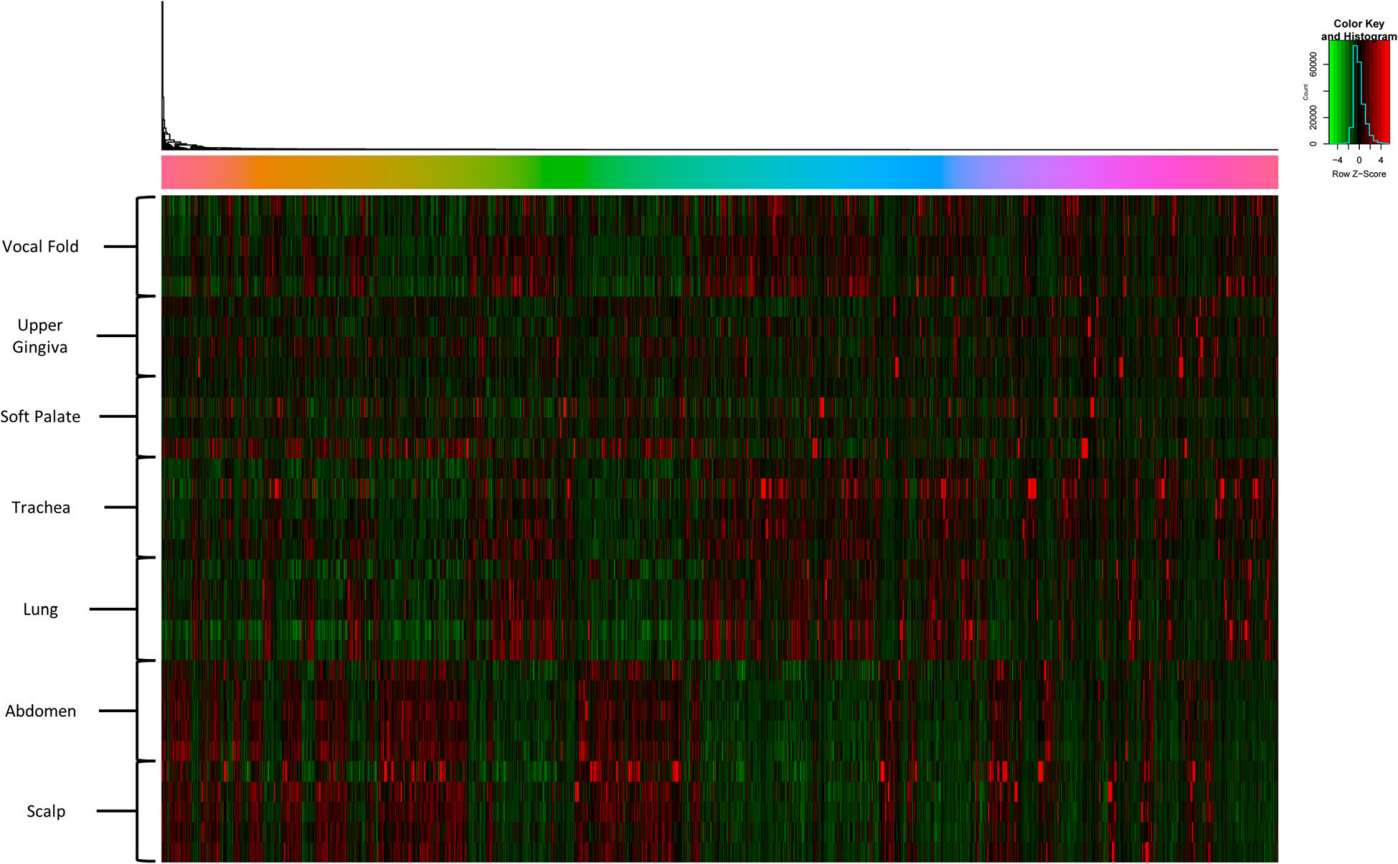
Transcriptomic heatmap exhibiting 6216 genes differentially expressed across all seven anatomic locations. Differential gene expression pattern analysis for vocal fold versus upper gingiva versus soft palate versus trachea versus lung versus abdomen versus scalp dermis identified by RNA sequencing. Adjusted *P* < 0.05. Rainbow colored dendrogram panel represents clustering of genes, where closely related genes will be grouped together. Genes within a cluster are in a similar color and more correlated to each other than to genes outside that cluster

**Fig. 4:**
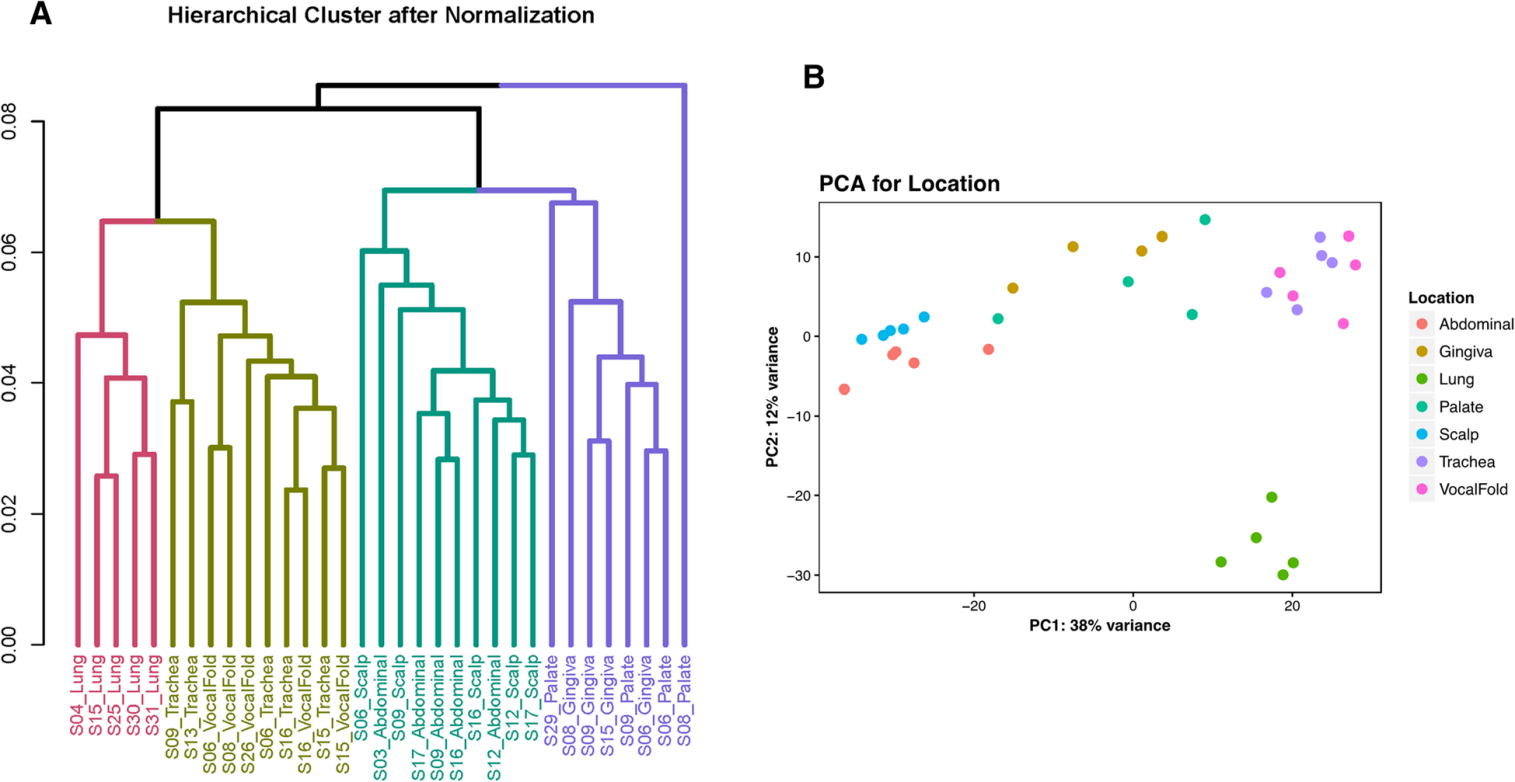
Human fibroblasts display phenomenon of global topographic differentiation. **a** Hierarchical clustering plot for all samples exhibiting similarity in global gene expression profiles across 33 total samples. **b** Principle component analysis (PCA) plot for 33 total samples by location

Acquisition of gene expression profiles were obtained by next generation RNA sequencing of the entire genome which yielded 6216 differentially expressed (DE) genes in total across all cell type comparisons (e.g. vocal fold versus trachea versus lung versus abdomen versus scalp versus upper gingiva versus soft palate) with the False Discovery Rate (FDR) corrected at 5% (Fig. 3). Unsupervised hierarchical cluster analysis of the data revealed fibroblast grouping based on anatomic site origin (e.g. vocal fold, trachea, lung, abdomen, scalp, upper gingiva, soft palate) rather than cells from the same donor (Fig. 4a). Principle component analysis (PCA) for age- and sex-related effects were negligible between groups (Fig. 4b).

### Post-hoc differential expression

Separate post-hoc 2-group comparative analyses for transcript-level differential expression were performed for: (1) vocal fold versus trachea, (2) vocal fold versus lung, (3) vocal fold versus upper gingiva, and (4) vocal fold versus soft palate fibroblast cell types. These comparisons were chosen based on our preliminary data (see Fig. 4a) which exhibited similar phylogenetic familial relationships between vocal fold, trachea, and lung cell types. Further investigation may provide evidence for demarcation of gene sets which all derive from anterior foregut endoderm, thereby improving insight into developmental domains as it relates to the VFF genotype. In addition, upper gingiva and soft palate comparisons were chosen to provide data alongside cell types known for minimal scar formation, thereby increasing our understanding into the molecular crosstalk, which may help direct gene-targeted cellular engineering for optimal VF remodeling paradigms. Highly differentially expressed genes across all post-hoc 2-group comparisons, as well as, data not discussed are included as additional files [see Additional file 1 and Additional file 2].

### Vocal fold versus trachea differential expression

**Fig. 5:**
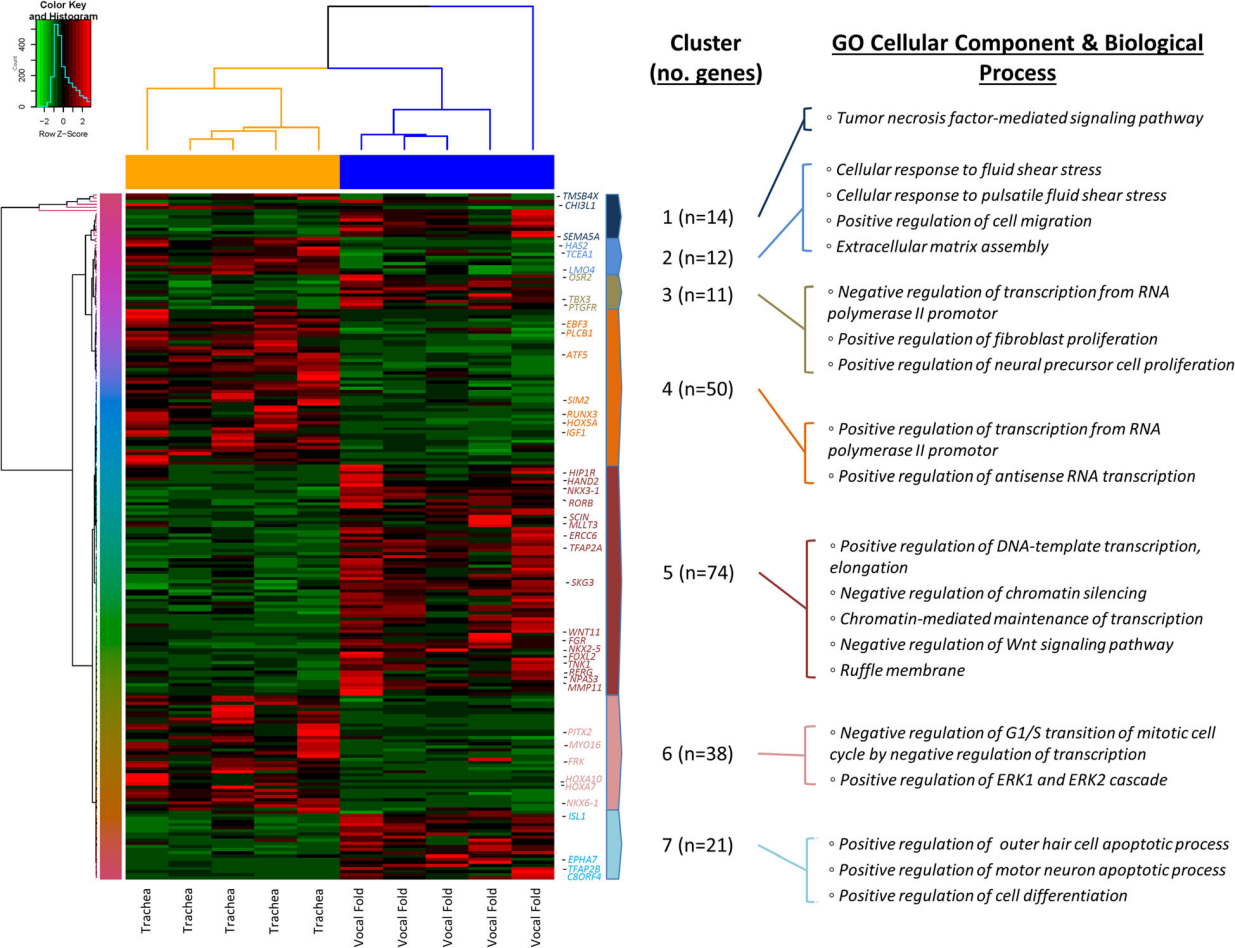
Differential gene expression pattern analysis for vocal fold versus trachea identified by RNA sequencing. Transcriptomic heatmap exhibiting clustering of 220 genes differentially expressed (116 were upregulated (red) and 104 were downregulated (green) in the vocal fold condition; Adjusted *P* < 0.05). Most highly expressed genes with their associated overrepresented biologic terms are indicated within each cluster pattern (right). Rainbow colored dendrogram panel represents clustering of genes, where closely related genes will be grouped together. Genes within a cluster are in a similar color and more correlated to each other than to genes outside that cluster

Supervised gene expression analysis yielded 220 genes differentially expressed in vocal fold versus trachea, of which, 116 were upregulated and 104 were downregulated in the vocal fold condition (Fig. 5).

Further refinement and greater biological characterization of gene sets were pursued through functional enrichment analyses utilizing Enrichr software. Comparative analysis identified significant GO biological process terms within the upregulated vocal fold condition associated with positive regulation of DNA-templated transcription, elongation; negative regulation of chromatin silencing; and chromatin-mediated maintenance of transcription *(NKX3–1, NKX2–5, TFAP2A, WNT11, HAND2, FOXL2, NPAS3, RORB, ERCC6)*, negative regulation of canonical Wnt signaling pathway involved in controlling type B pancreatic cell proliferation *(TFAP2A, WNT11, SCIN, PTPRO, MLLT3, NKX2–5, RERG, NKX3–1)*, positive regulation of antisense RNA transcription; positive regulation of mating-type specific transcription, DNA-templated; positive regulation of transcription during mitotic cell cycle *(TFAP2A, OSR2, TFAP2B, WNT11, HAND2, TP53INP2, FOXL2, RORB, NKX2–5, NPAS3, NKX3–1),* positive regulation of cell proliferation *(FGR, OSR2, TFAP2B, TNK1, TNFRSF11B, NKX2–5, NKX3–1, PTGFR, S1PR3, SGK3, TBX3)* and differentiation *(ISL1, TFAP2B).* Several functional biologic themes were appreciated related to transcription factors as well as extracellular matrix components such as cell signaling communication, migration, proliferation, and differentiation, in large part, driven by combinatorial gene transcripts within the fifth cluster of our transcriptomic heatmap. Additionally, within this cluster, we found a significant GO cellular component term associated with ruffle membrane *(FGR, HIP1R)*, which represents the structural changes on a motile cell surface that contains a lattice of newly polymerized actin filaments. *MMP11* was also found to be upregulated within this cluster. Surprisingly, *HAS2*, which has important implications for cell migration and response to fluid and pulsatile shear stress was found to be downregulated in vocal folds compared to trachea fibroblast cell type.

Furthermore, DE genes upregulated in the vocal fold condition were subjected to analysis using KEGG and WikiPathways cell signaling pathway databases. KEGG analysis generated significant terms associated with axon guidance *(SEMA5A, EPHA7, UNC5C, UNC5D, NTN1)*, tyrosine metabolism (*ALDH3A1*, *ALDH3B1*) as well as overlapping genes of *TBX3, ISL1*, and *WNT11* significant for signaling pathways regulating pluripotency of stem cells. WikiPathways analysis yielded significant terms associated with heart development *(HAND2, NKX2–5, ISL1)*, preimplantation embryo *(TFAP2B, TBX3, NR3C2)*, and neural crest differentiation *(TFAP2A, TFAP2B, ISL1)*.

### Vocal fold versus lung differential expression

**Fig. 6:**
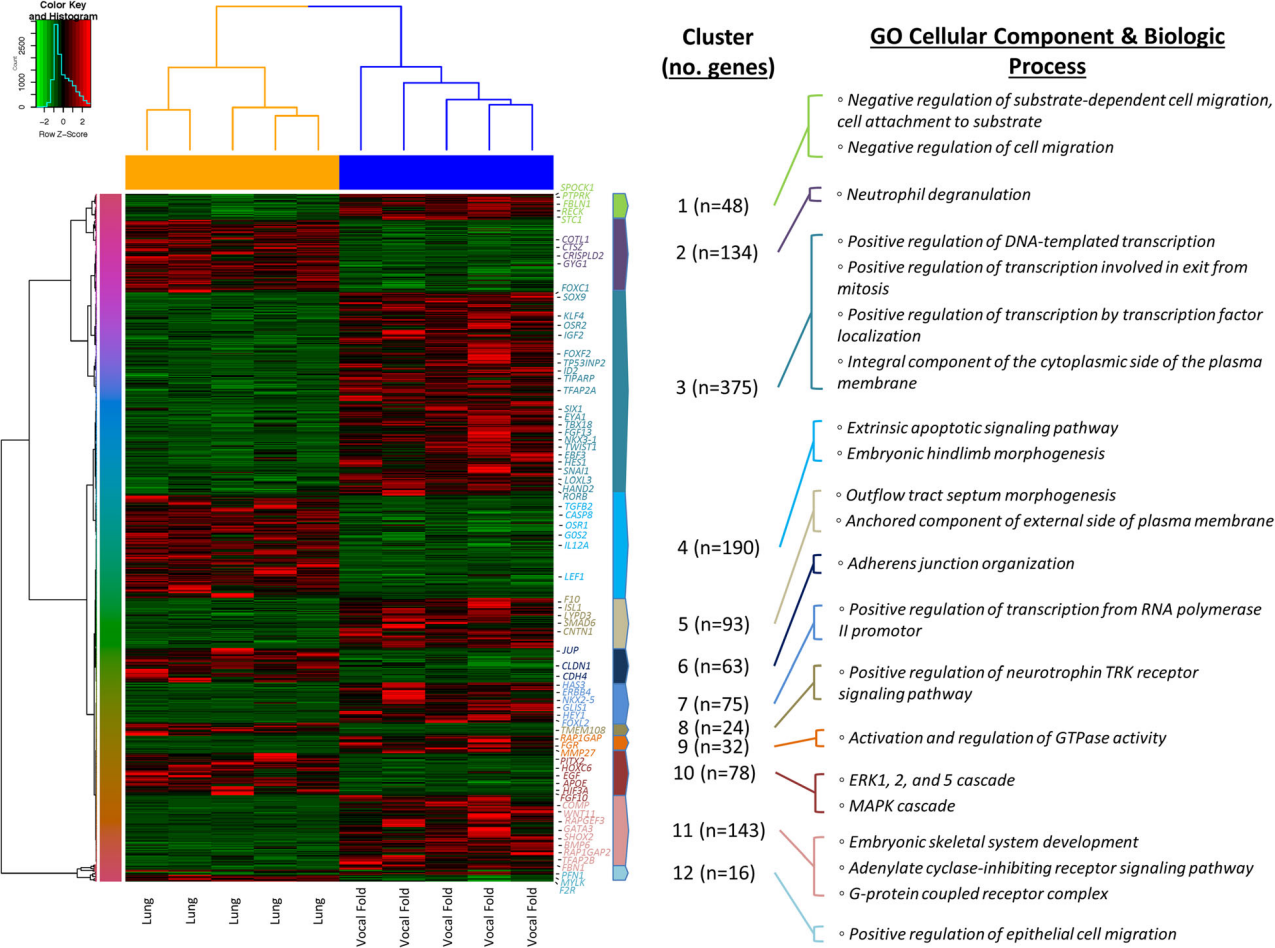
Differential gene expression pattern analysis for vocal fold versus lung identified by RNA sequencing. Transcriptomic heatmap exhibiting clustering of 1271 genes differentially expressed (766 were upregulated (red) and 505 were downregulated (green) in the vocal fold condition; Adjusted *P* < 0.05). Most highly expressed genes with their associated overrepresented biologic terms are indicated within each cluster pattern (right). Rainbow colored dendrogram panel represents clustering of genes, where closely related genes will be grouped together. Genes within a cluster are in a similar color and more correlated to each other than to genes outside that cluster

Vocal fold versus lung fibroblast cell types yielded 1271 genes differentially expressed, of which, 766 were upregulated and 505 were downregulated in the vocal fold condition (Fig. 6).

Significant GO biological process terms within the upregulated vocal fold condition were motivated by combinatorial gene transcripts within the third, seventh, and eleventh clusters of our transcriptomic heatmap. Enriched biologic terms identified within cluster 3, in order of significance, were associated with positive regulation of transcription involved in exit from mitosis (*HDAC4, TFAP2A, OSR2, FOXC1, PCID2, FOXF2, SIX1, EBF3, RORB, KLF4, SMARCA2, NPAS2, PIAS2, EPS8, ING2, TP53INP2, HAND2, ID2, ATOH8, SNAI1, SOX9, CDK5RAP2, NKX3–1),* positive regulation of DNA-templated transcription for processes of initiation, elongation, and termination *(HDAC4, FOXC1, SIX1, GATA3, RORB, NPAS2, ING2, WNT11, SFR1, ERBB4, TP53INP2, PRDM16, ATOH8, HAS3, ALX1, SOX9, NKX2–5, ZNF423, CDK5RAP2, NKX3–1, TFAP2A, NFE2, TFAP2B, OSR2, PCID2, FOXF2, NFATC1, FOXL2, EBF3, KLF4, RGMB, SMARCA2, PIAS2, MDFIC, IRF4, HAND2, ID2, SNAI1, ERCC6),* and positive regulation of transcription by transcription factor localization (*HDAC4, TFAP2A, OSR2, FOXC1, PCID2, FOXF2, LRRK2, SIX1, EBF3, RORB, KLF4, SMARCA2, NPAS2, PIAS2, ING2, TP53INP2, HAND2, ID2, ATOH8, SNAI1, SOX9, CDK5RAP2, NKX3–1).* Enriched biologic terms identified within cluster 7 were associated with positive regulation of transcription from RNA polymerase II promotor *(NFE2, HEY1, ERBB4, IRF4, KCNIP3, HNF4G, HAS3, FOXL2, NKX2–5, GLIS1)* as well as cluster 11 associated with embryonic skeletal system development *(COMP, WNT11, SHOX2, TNFRSF11B, ARSE, BMP6, FBN1),* and adenylate cyclase-inhibiting G-protein coupled receptor signaling pathway *(GNAL, APLP1, ADCY4, LPAR1, ADCY1).* Additionally, *HAS3* was found to be upregulated in multiple biologic processes within cluster 7, *MMP27* was upregulated within cluster 9, and *RAP1GAP* and *RAP1GAP2* were found to be upregulated within cluster 9 and 11, respectively. Most highly represented GO cellular component terms within the upregulated vocal fold condition were found to be associated with integral component of external side of the plasma membrane, insulin receptor complex, integral component of the cytoplasmic side of the plasma membrane, and the G-protein coupled receptor complex.

KEGG pathway analysis yielded significant activation of the calcium signaling pathway *(PTGFR, PTGER3, ADCY4, ITPR3, ADRB1, ADCY1, TACR1, CYSLTR1, GRIN2A, HRH1, GNAL, CCKAR, EDNRB, PHKG1, STIM2, ERBB4, BDKRB2, PLCE1),* Rap1 signaling pathway *(MAGI3, ADCY4, LPAR1, ADCY1, RAP1GAP, FYB, IGF1R, APBB1IP, EFNA1, TIAM1, GRIN2A, SIPA1L1, ID1, PLCE1, FGF13, DRD2, RAPGEF3),* and cAMP signaling pathway *(VAV3, PTGER3, ADCY4, NFATC1, ADRB1, ADCY1, MAPK10, TIAM1, GRIN2A, PLCE1, SOX9, DRD2, RAPGEF3, GRIA3);* while WikiPathways generated terms identical to our vocal fold versus trachea enrichment analysis, however, with the addition of significant prostaglandin synthesis and regulation *(PTGFR, PTGIS, EDNRB, PTGER3, PLA2G4A, PTGS2, PTGS1, PTGDR),* G-protein signaling pathway *(AKAP12, GNAL, ADCY4, ADCY1)* as well as ectoderm differentiation *(TFAP2A, GRAMD1B, NLGN1, ELOVL4, CTNND2, FZD8, STC1, NFATC1, TNFRSF11B, POU2F2, PTPN13, ZFHX4, TSKU, PLCXD3, PODXL, ANKS1B).*

### Vocal fold versus upper gingiva differential expression

**Fig. 7:**
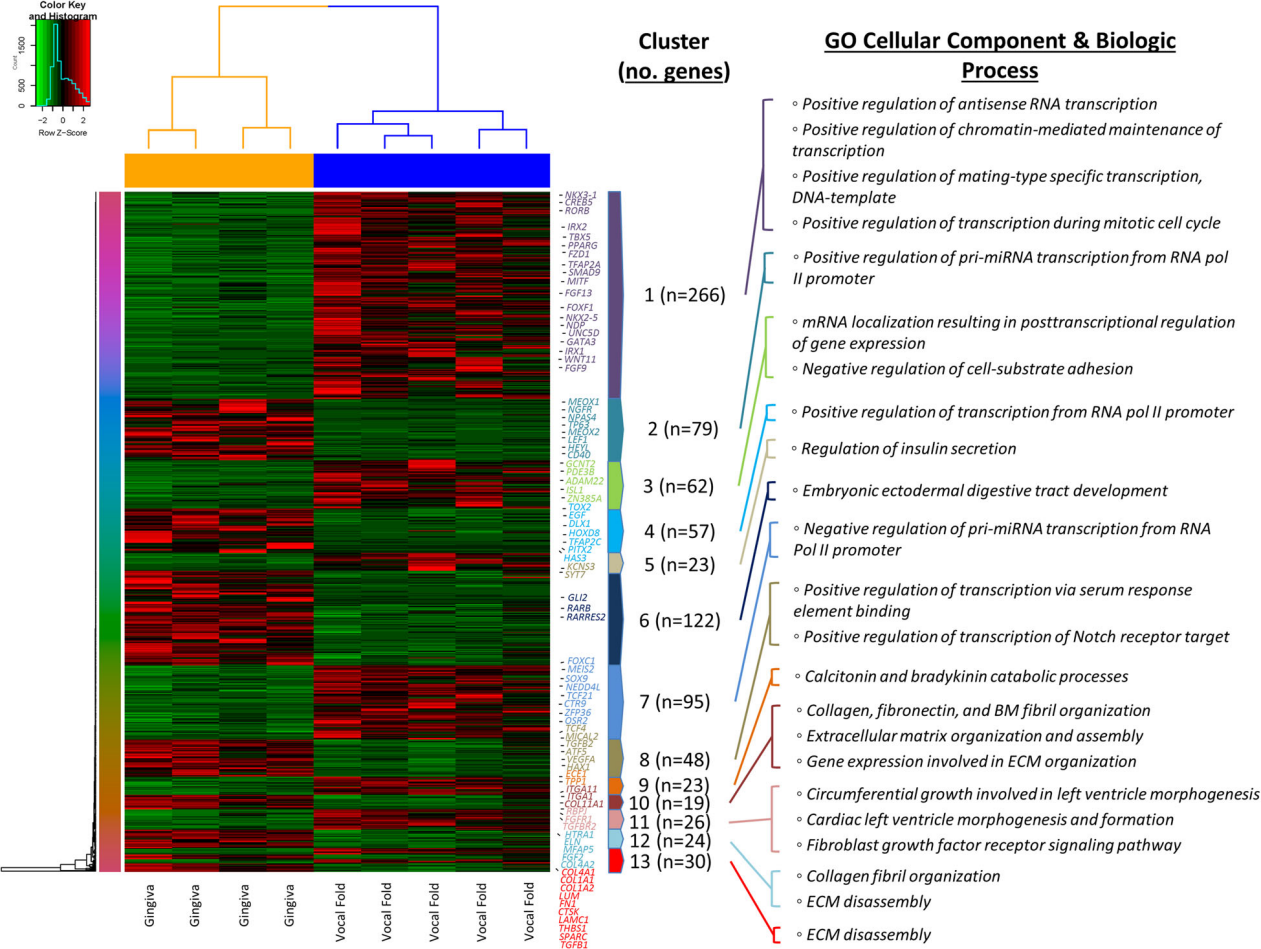
Differential gene expression pattern analysis for vocal fold versus upper gingiva identified by RNA sequencing. Transcriptomic heatmap exhibiting clustering of 874 genes differentially expressed (508 were upregulated (red) and 366 were downregulated (green) in the vocal fold condition; Adjusted *P* < 0.05). Most highly expressed genes with their associated overrepresented biologic terms are indicated within each cluster pattern (right). Rainbow colored dendrogram panel represents clustering of genes, where closely related genes will be grouped together. Genes within a cluster are in a similar color and more correlated to each other than to genes outside that cluster

Vocal fold versus upper gingiva fibroblast cell types yielded 874 genes differentially expressed, of which, 508 were upregulated and 366 were downregulated in the vocal fold condition (Fig. 7).

Significant GO biological process terms within the upregulated vocal fold condition were again motivated by multiple transcriptional regulation processes within cluster 1, however, more interesting was the highly represented GO cellular component terms which identified significance associated with integral components of the internal and external plasma membrane as well as transforming growth factor beta receptor complex *(LPAR1, PCDH18, TM4SF1, TGFBR2, FGFR1)* within cluster 1 and 11, respectively.

KEGG pathway analysis also generated significant activation of the Rap1 signaling pathway and pathways in cancer *(FZD1, LAMA5, HGF, ADCY4, LPAR1, MITF, PIK3R1, PIAS2, TGFBR2, MAPK10, AR, WNT11, MECOM, FGF9, LPAR6, KIT, PPARG, FGF13, WNT2, NKX3–1, FGFR1)*; while WikiPathways yielded significant activation of the Wnt signaling pathway *(MAPK10, FZD1, PRKCI, WNT11, WNT2, ROR2, PRKCH)* and small ligand GPCRs *(PTGFR, LPAR1, S1PR1, S1PR3).*

### Vocal fold versus soft palate differential expression

**Fig. 8:**
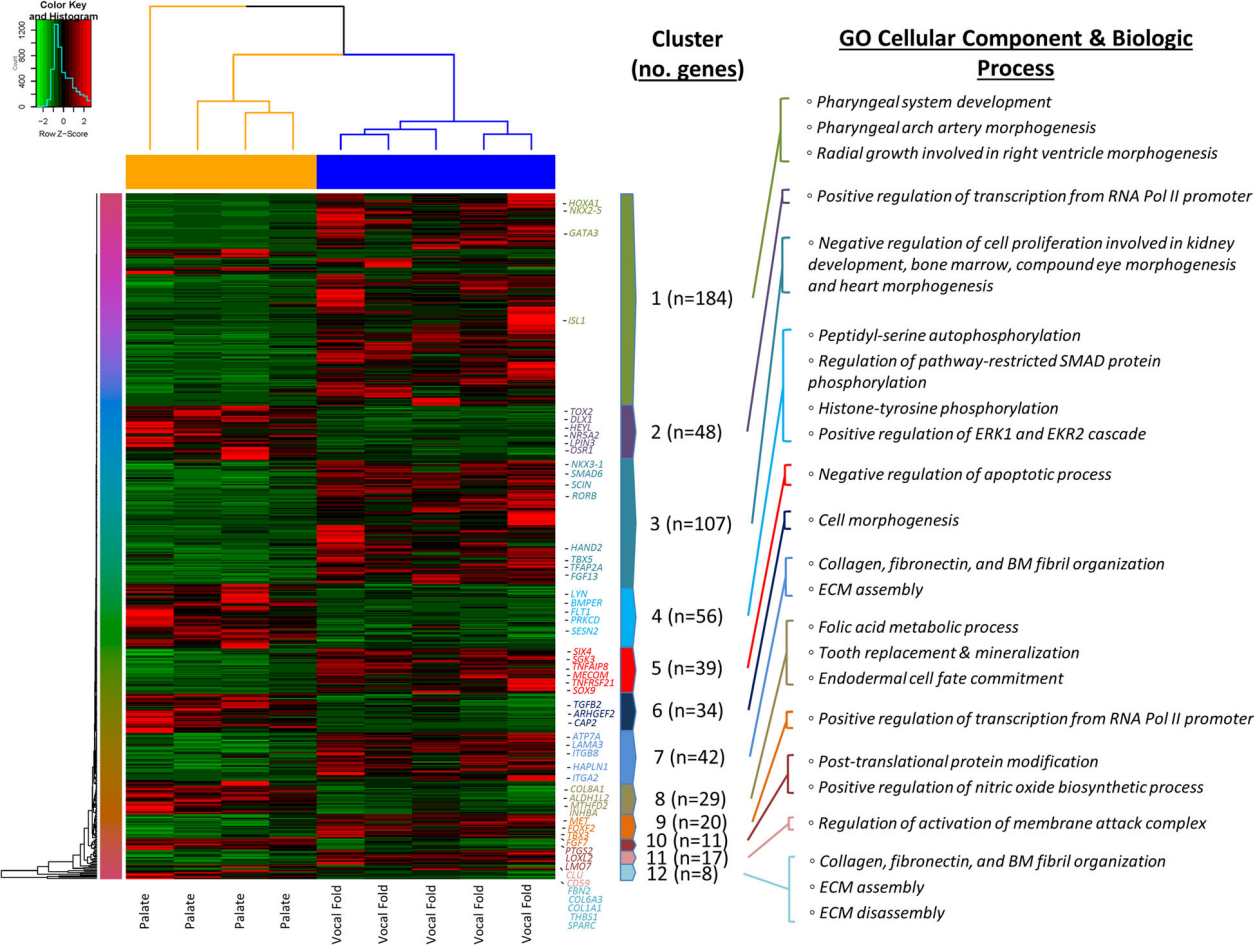
Differential gene expression pattern analysis for vocal fold versus soft palate identified by RNA sequencing. Transcriptomic heatmap exhibiting clustering of 595 genes differentially expressed (393 were upregulated (red) and 202 were downregulated (green) in the vocal fold condition; Adjusted *P* < 0.05). Most highly expressed genes with their associated overrepresented biologic terms are indicated within each cluster pattern (right). Rainbow colored dendrogram panel represents clustering of genes, where closely related genes will be grouped together. Genes within a cluster are in a similar color and more correlated to each other than to genes outside that cluster

Vocal fold versus soft palate fibroblast cell types yielded 595 genes differentially expressed, of which, 393 were upregulated and 202 were downregulated in the vocal fold condition (Fig. 8).

Significant GO biological process terms within the upregulated vocal fold condition were motivated by combinatorial gene transcripts within clusters 1, 3, 5, and 11 of our transcriptomic heatmap. Data revealed that the top three most highly significant terms were associated with negative regulation of epithelial cell proliferation *(TFAP2A, TFAP2B, CDKN2A, HMGA1, GATA3, SMAD6, TBX5, TOB1, RERG, SCIN, SIX4, ADAMTS1, ZNF503, SOX9, ROR2, IGFBP6, DRD2, ADAMTS8, NKX3–1),* negative regulation of cell proliferation *(TFAP2A, TFAP2B, CDKN2A, HMGA1, GATA3, TBX5, SMAD6, TOB1, AGT, RERG, SCIN, ADAMTS1, ZNF503, SGK3, ROR2, IGFBP6, SOX9, DRD2, ADAMTS8, TNFRSF21, NKX3–1),* and negative regulation of hemocyte proliferation *(TFAP2A, TFAP2B, CDKN2A, HMGA1, GATA3, SMAD6, TBX5, TOB1, RERG, SCIN, ADAMTS1, ZNF503, SOX9, ROR2, IGFBP6, DRD2, ADAMTS8, NKX3–1).* Significant GO cellular component terms were found to be related to integral component of the cytoplasmic side of the plasma membrane as well as the membrane attack complex, which represents a protein complex produced by the complement cascade which inserts into a target cell membrane and forms pores resulting in cell lysis via ion and water flow.

KEGG pathway analysis yielded the calcium signaling pathway, dopaminergic synapse, and axon guidance as the top three most highly represented associated pathways; while WikiPathways generated similar activation pathways of preimplantation embryo *(FOXQ1, TFAP2B, IRF4, HMGA1, GATA3, AQP3, ZFP36L2, TBX3, NR3C2),* heart development *(FOXC1, HAND2, TBX5, NKX2–5, ISL1),* neural crest differentiation *(TFAP2A, TFAP2B, ID1, HOXA1, SOX9, ISL1, SOX5),* however, with the addition of cardiac progenitor differentiation *(ROR2, NKX2–5, TBX5, DKK1, ISL1).*

## Discussion

Unsupervised hierarchical cluster analysis of the data revealed grouping based on anatomic site origin rather than donor, suggesting global fibroblast phenotype heterogeneity and which corroborates previous transcriptomic work [6–8]. Contrary to previous work that has demonstrated sex-dependent [21] and age-related effects [22–25] on gene expression, our results were negligible. However, sex differences in many traits are often subtle which require large sample cohorts for sufficient power in revealing discrepancies [21], which may explain our lack of significance. The absence of age-related effects were also surprising, albeit, prior research suggests highly varied data even among comparable aged individuals likely related to genetic factors, lifestyle, and/or environmental exposures. Our lack of age-related differences may be of benefit to our analyses as epigenetic regulation impacts gene expression [22, 23]. In view of this, our data, however, established clear delineations of fibroblast gene expression programs observed across tissue-specific anatomic locales and elucidated the unique genotype and cognate biologic and cellular functions of the VFF; a cell-type which undergoes significant and extensive mechanical loading forces.

### Fibroblasts under high mechanical demands exhibit unique functional roles primed for cellular plasticity

Fibroblasts play a vital role in tissue function, normal tissue morphology, and mechanical support for tissues [20]. Previous research has shown that fibroblasts can be driven to multipotency under strict biochemical cues and mechanical loads [26]. Furthermore, fibroblasts exhibit cellular plasticity with the unique ability to alter their phenotype for occupation of varying functional roles [27–29]. Although less understood, we are beginning to appreciate the heterologous molecular response of fibroblasts that undergo varying mechanical stress [2, 3, 9, 16, 17]. We chose to use the VFF as our surrogate cell type due to the unique biomechanical demands of this organ and tissue-type [10, 18, 19]. Our overarching goal was to utilize this cell-surrogate to identify mechano-responsive genes coding for biologic and cellular mechanisms that may contribute to extracellular matrix homeostasis.

Results demonstrated global demarcation of the fibroblast cell type across all anatomic site domains with the specialized genotype of the VFF uniquely characterized to achieve homeostasis under complex mechanobiological requirements. In specific, we found increased expression of gene transcripts *FGR* and *HIP1R,* in the vocal fold versus trachea comparative analysis, which were associated with the GO cellular component of cell membrane ruffling. This structural phenomenon has been associated with Rho-GTPase activity and is observed as the formation of actin-rich membrane protrusions on the cell surface, which provide a unique mechanism for targeted molecular transduction, critical for regulation of immune response, cytoskeleton remodeling in response to extracellular stimuli, and cell motility [30–33]. To our knowledge, this is the first study that has identified specific genes involved in this unique membranous cellular process within the VFF which may have acquired to rapidly ameliorate remodeling of the ECM composition. Additionally, we found significant GO cellular component, biologic process, and signaling pathway terms, within the vocal fold versus lung comparative analysis, associated with activation and regulation of GTPase activity, G-protein coupled receptor complex, Rap1 and cAMP signaling pathways. These pathways seem to be driven by the genes *Rap1GAP* and *Rap1GAP2*. These gene homologues encodes a type of GTPase-activating protein (GAP) that down-regulates the activity of the Ras-related *Rap1* protein which is well-known for its role as a molecular switch, cycling between inactive GDP-bound and active GTP-bound conditions [34]. *Rap1GAP*, therefore, acts to inhibit *Rap1* activity, resulting in a cellular “protective mechanism” by inhibiting proliferation, impairing cell invasion and metastasis, and accelerating apoptosis [35–40]. Furthermore, *Rap1GAP* has also been recognized for its role on impairing cell-matrix adhesion in the absence of effects on cell-cell adhesion [41].

Aiding in ECM regulatory processes are the presence of MMPs, vital for cell migration and degradation of cellular debris. Our results found that *MMP11* was upregulated in VFF compared to trachea as well as *MMP27* upregulated in VFF compared to lung cell comparisons. Another interesting observation was the upregulation of *IGF2* and *IGF1R* genes within the VFF compared to lung cell-type comparison. *IGF2* encodes a member of the insulin family of growth factors involved in development and growth, whereas, *IGF1R* is a high-affinity receptor for insulin-like growth factors with its ligands involving *IGF1*, *IGF2*, and insulin [42]. Additionally, recent work has elucidated biochemical mechanistic interactions involving Rap1 signaling by presumed *Rap1GAP* and *IGF1R* [43]. Taken together, it seems that the VFF, under constant mechanical forces, may have developed dynamic genetic regulatory mechanisms for active motility, and close surveillance of the surrounding extracellular environment.

Another important pathway expressed by VFFs, which aid in regulating the local, bioactive milieu seem to be genes associated with prostaglandin synthesis and regulation *(PTGFR, PTGIS, EDNRB, PTGER3, PLA2G4A, PTGS2, PTGS1, PTGDR*). These gene transcripts are variants for prostaglandin receptors of the G-protein coupled receptor family (*PTGFR*, *PTGDR, PTGER3),* important for binding affinity to their respective ligands for inflammatory regulation and synthesis (*PTGIS, PTGS1*, *PTGS2).* Previous work has shown the complexity of prostaglandin E2 (*PGE*_*2*_), as both a regulator of inflammation and agonist of healing during the proliferative phase through its interactions with mesenchymal cells enlisted into the wound bed [44]. Prostaglandin-endoperoxide synthase 2 (*PTGS2),* otherwise known as *COX-2,* is the downstream product of *PGE*_*2*_*,* and has been implicated in immune mediation of inflammation; however seems to be organ-dependent with regard to function [45–49]. In vitro research investigating signaling of immortalized VFFs found that interleukin 1 beta (*IL-1β*) regulated *COX-2* in a dose- and time-dependent manner, altering *PGE*_*2*_ metabolism by upstream translocation of nuclear factor (NF)-кB [49]. Due to the elusive attributes of *PGE*_*2*_, this same group sought out to determine the effects of *PGE*_*2*_ on VFF and its interaction with *TGFβ-1.* Results revealed that *PGE*_*2*_ does have an antifibrotic effect on the VFF, however, that exogenous *TGFβ-1* elicits induction of *COX-2*, suggesting inherent pathway signaling complexity [44]. Our data did exhibit upregulation of *COX-2* in both VF versus lung and VF versus palate comparisons; however, a lack of identified overexpressed *PGE*_*2*_ within the VFF was evident across all other 2-group cell type comparisons*.* Given this we speculate that upon insult, crosstalk between *TGFβ-1* and *COX-2* strengthens, enhancing *COX-2* expression, which results in increased *PGE*_*2*_ expression thereby dampening profibrotic fibroblast activity in vivo. Additional studies are needed to fully appreciate this complex signaling pathway. Our results suggest that mechano-responsive fibroblasts may retain low levels of chronic inflammation under their respective conditions, albeit in a controlled manner for ongoing homeostasis.

Equipoise between profibrotic and antifibrotic fibroblast phenotype is critical for maintaining extracellular homeostasis. Aiding in mechanical support for active tissue-types are a family of enzymes entitled, hyaluronic acid synthases (HAS). This gene family consists of three genetic homologues (e.g. *HAS1*, *HAS2, HAS3*) that code for the hyaluronic acid (HA) protein found in high concentrations in diverse connective tissues and serves to provide ECM stability, viscoelasticity, hydration, shock absorption, and movement of substances [50]. Characterization of HAS enzymes have identified two categories based on molecular weight and which correspond to differing physiological functions. Specifically, *HAS1* and *HAS2* produce high molecular weight HA important for cellular adhesion and inhibition of cellular proliferation. *HAS3,* on the other hand, produces low molecular weight HA critical for initiating signaling cascades, stimulating cell proliferation, angiogenesis, and inflammation [50–54]. Our data exhibited decreased expression of *HAS2* in VFF compared to trachea, however, increased expression of *HAS3* in VFF compared to lung, albeit decreased expression of *HAS3* in VFF compared to gingiva fibroblast. While the former is divergent to what was expected, previous research has established the importance of HA in determining the viscoelastic properties of the vocal fold cover as well as the role played under normal physiologic conditions and within wound-healing paradigms [55–57]. Our varied results regarding *HAS3* expression across different cell type comparisons reinforce its tissue-dependent function. These results also conform previous characterization work with regard to increased *HAS3* expression within cell types well-known for rapid cell proliferation upon wounding and increased ability for minimal scar formation [58–60]. Overall, our findings exhibit decreased expression of *HAS2*, increased expression of *HAS3*, increased biologic terms associated with cell proliferation, and multiple active signaling cascades (e.g. Rap1, cAMP, prostaglandin synthesis and regulation).

Taken together, our data represent intriguing evidence for manners in which fibroblasts respond to support frequent and high inertial stresses. Previous literature draws parallels to our findings, exhibiting Rap1 as a major signaling cascade pathway in the VFF genotype with coordination from insulin growth factors, prostaglandins, and MMPs for targeted cellular and extracellular environmental monitoring. Furthermore, presumed mechanical forces on the vocal fold tissue niche seem to engender a uniquely characterized VFF genotype, resulting in a phenotype inclined for immediate response to local environmental fluctuations via signaling pathways and active ECM remodeling under homeostatic conditions; thus avoiding more severe ensuing inflammatory consequences.

### VFF lack disassembly mechanisms established in minimal scarring tissue phenotypes

Currently and within the field of laryngology, effective remedial treatment options are inadequate for VF scarring of the lamina propria. Therefore, comprehensive characterization of genetic cues contributing to (or lack thereof) the wound healing paradigm are necessary for advancing the field. Due to these clinical shortcomings, we performed comparative analyses involving the VFF against oral mucosa tissue types well known for minimal scarring (e.g. soft palate fibroblast, upper gingiva fibroblast). Enrichment analysis identified two new functional themes upregulated within the VFF compared to the soft palate and upper gingiva fibroblast, which included the cellular component term associated with membrane attack complex (*CD59*, *CLU*), and transforming growth factor beta receptor complex *(LPAR1, PCDH18, TM4SF1, TGFBR2, FGFR1),* respectively.

The membrane attack complex (MAC), is a multi-protein pore found inserted on cell membranes, which upon activation inserts into and directly lyses microbes and a wide range of Gram-negative bacteria essential for innate immune host defense [61, 62]. *CD59* (also known as *protectin*) encodes a glycoprotein on the cell surface that regulates complement-mediated cell lysis by inhibition of MAC. While the overexpression of this protein protects against circulating complement, increased internalization has been correlated with hypoxic conditions and results in endothelial cell damage [63, 64]. *CLU* (also known as *clusterin*) encodes for numerous homologous chaperone proteins whose functions depend upon topological location, with variants aiding in prevention of nonnative protein aggregates [65], cellular apoptotic signaling, and inhibiting cell growth and survival [66, 67]. Furthermore, recent evidence found that laminar shear stress negates endothelial cell activation through upregulation of *CLU*, inhibiting the proinflammatory response by reduced expression of interleukin-8 (IL-8) and monocyte chemoattractant protein-1 (MCP-1). In contrast, expression of *CD59* was not found to be affected by shear stress [68]. To date, there is a paucity of literature characterizing the laryngeal microbiome and immunologic bionetwork, specifically, within the vocal fold cellular and extracellular milieu, and to our knowledge this is the first study to identify this unique cell membrane function in the VFF.

Since we were also interested in gene transcripts upregulated within oral mucosa, which may provide insight into its advanced wound-healing properties, we explored reciprocal enrichment analysis and found significant GO biologic process terms associated with ECM disassembly across both soft palate and upper gingiva fibroblast genotypes compared to VFF. Transcripts encoding for ECM disassembly involved adhesive structural proteins (*COL1A1, COL1A2, COL4A1, COL6A3, FBN2, FN1, LAMC1),* supporting matricellular proteins (*SPARC, THBS1)*, as well as, proteoglycan *(LUM)* and proteinase *(CTSK)* transcripts [see Additional file 3]. *COL1A1, SPARC,* and *THBS1* were identified to be upregulated with overlap across both soft palate and upper gingiva comparisons*.* Previous research has elucidated that expression of *SPARC* (otherwise known as *osteonectin*) functions to regulate cell-matrix interactions influencing numerous important physiological and pathological processes. Specifically, in adult stages, *SPARC* function is largely limited to tissue remodeling during homeostasis and wound healing both through its growth factor modulatory activity (e.g. PDGF, VEGF, bFGF), cell cycle-inhibition, and de-adhesive properties via altering cell morphology [69, 70]. Research investigating VF tissue, identified genes differently expressed in polyp lesions versus Reinke’s edema, and found *SPARC* significantly upregulated in VF polyps, suggesting its reparative role in regulating the polyp microenvironment [71]. However, *SPARC* has also been implicated in oncologic studies playing a prominent role in both tumorigenesis and tumor suppression [72]. Thrombospondin 1 *(THBS1),* another matricellular supportive protein, is a potent angiogenesis inhibitor and has been found to dictate wound healing as well as prevention of tumor progression through its participation with p53 [73, 74]. Although previous research have uncovered the wide diversity of matricellular protein contribution to normal and oncologic biology, much is still unknown about the pathophysiology of this unique group of modular ECM proteins, with our data establishing its increased expression during homeostasis in the oral mucosa microenvironment.

Another interesting finding were the identification of gene transcripts *LUM* (otherwise known as *lumican*) and *CTSK* (otherwise known as *cathepsin K*) associated with the GO biologic process term of ECM disassembly, albeit, upregulated within the VFF compared to both soft palate and upper gingiva fibroblast cell types. *CTSK* encodes a lysosomal cysteine proteinase and is essential for normal bone resorption and remodeling [75], whereas, *LUM* encodes for a small leucine-rich proteoglycan (SLRP) that has been shown to regulate collagen fibrillogenesis via modulation of epithelial cell adhesion and migration, in addition to, its recently discovered role for wound healing within the corneal epithelium [76]. SLRPs are not only well-known for modulating a variety of cellular behaviors, including migration, proliferation, and tissue repair, but also regulation of the ECM environment via its effect on tissue hydration [77, 78]. This finding is important for vocal fold biology as decorin, another SLRP, has been previously described predominating in the superficial layer of the lamina propria; functioning to decrease collagen fiber size and density in addition to its ameliorating effect on tissue injury and healing [79]. Speculation has also been given to the effects decorin has on fibroblastic response to tissue injury [80]. This data adds further evidence of the increased diverse presence of SLRPs within the vocal fold milieu, which may play a larger role than previously recognized with regard to tissue architecture and cellular remodeling.

Previous research has also identified *SPARC* to orchestrate with transforming growth factor beta-1 (*TGFβ-1)* in human fibroblasts*,* with increased *TGFβ-1* mRNA expression paralleled to increased *SPARC* synthesis during wound healing [81]. Furthermore, mutual upregulated expression of lysyl oxidase-like 2 *(LOXL2)* was found in both oral mucosa cell types, with prior research demonstrating this cross-linking enzyme as a critical regulator of ECM organization by enhancing TGF*β* signaling [82]. Our data supports these findings and exhibits that these genetic transcripts (*TGFβ-1, TGFβ-2)* seem to also associate during homeostasis in rapidly renewing tissue types such as oral mucosa. Given what we know about oral mucosa minimal scar phenotypes, and its active expression of *TGFβ* ligands in association with matricellular proteins and crosslinking-enzymes, we can assume that these cellular expression/collaborations are advantageous to the surrounding ECM in its ability for ongoing remodeling.

By performing comparative analysis utilizing the VFF, a mechanically stressed cell surrogate, we begin to appreciate trends within our data related to the numerous fibroblast genotypes whose origins derive along the unified airway. Specifically, fibroblasts from various anatomic origins seem highly functionally differentiated to the demands of their local mechanical microenvironment. Fibroblast cell types within the oral mucosa (e.g. soft palate, upper gingiva), due to chronic environmental exposure, seem to have adapted a maturated advantage related to the increased ability to efficiently assemble and disassemble their respective ECM, of which the latter was absent from the VFF. These data may provide important gene targets for regulating biological processes involved in tissue development, growth, and regeneration and repair.

### VFF share multiple phylogenetic relationships with the developing heart

Recent work has shown that regardless of heterogeneous tissue types, adult stem cells preferentially express certain gene transcripts and share higher-order patterns of gene expression relating to specific intracellular processes (e.g. regulation of transcription, RNA binding, protein biosynthesis) [83]. Our global gene expression patterns exhibited reciprocity of multiple genes critical in heart development [see Additional file 3] and related processes with highly represented GO biologic process terms associated with DNA-templated transcriptional regulation processes of initiation, elongation, and termination, regulation of antisense RNA transcription as well as regulation of cell proliferation and differentiation. Highly upregulated gene transcripts identified were *HAND2, NKX2–5, ISL1, WNT11, OSR2, TBX3, TBX5, SOX9, SOX5, TFAP2A,* and *TFAP2B.*

It has long been known that craniofacial muscles are associated with head and neck structures and, in the developing embryo, these structures derive from the pharyngeal or branchial arches [84]. However, recent findings have recognized increased heterogeneity of muscle origins and progenitor fates of the vertebrate head [85–87], with emerging evidence suggesting a shared pool of mesoderm progenitor cells within the cardiopharyngeal field (CPF) of vertebrate embryos [88]. Our results identified numerous gene sets upregulated that are known to be involved in early developmental processes influencing site-specific cell fates. For example, *NKX2–5, HAND2, TBX3, ISL1, SIX1, EYA* and *HES* were upregulated in the VFF, overrepresented for numerous shared developmental origins related to regulation of head muscle structures (roof of mouth development, pharyngeal system development) and heart development and morphogenesis. In vertebrates, the CPF gives rise to the first-heart-field (FHF), the second-heart-field (SHF), and the branchiomeric muscles. Within the pharyngeal mesoderm, SHF progenitors produce the right ventricle, parts of the atria, and cardiac muscle tissue of the outflow tract [89–91]. Key players in this include the transcriptional factor *ISL1*, which marks a subset of CPF cells important for cardiovascular development and skeletal muscle progenitors [92], and *NKX2–5*, which regulates proliferation within the SHF and acts in concert with *ISL1* to modulate SHF progenitor-specific gene expression [93–95]. Coordinated clustering patterns were also appreciated between *NKX2–5* and *HAND2* across all cell type comparisons, which are known for their late stage embryonic differentiation capacity. *HAND2* belongs to the basic helix-loop-helix family of transcription factors and is known to play a key role in cardiac right ventricle and atria morphogenesis [96] within the SHF as well as limb and branchial arch development [97, 98]; while *NKX2–5* coordinates commitment to and/or differentiation of the myocardial lineage within the SHF. The presence of *NKX2–5* transcripts have also been implicated in other myogenic descendants within a murine model, such as, primitive pharyngeal endoderm, thyroid primordium, lingual, spleen, and stomach tissue with limited persistence into adulthood in some tissue types [99]. Targeted deletion assays have elucidated that the absence of endothelin-1 (ET-1), an enhancer controlling expression of *HAND2,* leads to cardiac abnormalities and a spectrum of craniofacial defects including cleft palate, cartilage malformation, and mandibular hypoplasia [99]. Another interesting finding was the overexpression of *TFAP2A,* and *TFAP2B,* alongside *ISL1* and various other CPF gene sets. These paralog gene transcripts encode for transcription factor proteins which have been implicated in vertebrate neural crest developmental and ectodermal evolution [100]. It has also been found that FGF signaling, specifically *FGF13*, plays a key role in neural development [101] with additional literature suggesting that FGF participation is necessary for *TFAP2A* pattern regulation indirectly via the Wnt-β-catenin pathway [102]. Interestingly, our data exhibited upregulation of *FGF13* in VFF across all cell type comparative analyses with the exclusion of the trachea fibroblast. Lastly, upregulation of multiple T-box genes were identified within the VFF across various cell type comparisons, which involved trachea *(TBX3),* lung *(TBX18),* upper gingiva *(TBX2, TBX4, TBX5)*, and soft palate *(TBX3, TBX4, TBX5).* Members of this highly conserved gene family encode for transcription factors responsible for regulation of developmental processes, and have been implicated in early heart development *(TBX2, TBX3, TBX5)* [103] as well as mammary gland development *(TBX2, TBX3)* [104]*,* with aberrant expression associated in tumorigenesis and abnormal development [104, 105]. Our data recognized numerous overexpressed TBX homologues within the VFF genotype and suggests a possible regulatory role in vocal fold development and specificity.

### Study limitations

Several limitations of this work warrant discussion. One limitation of this investigation was that we did not include other highly biomechanically sensitive tissues (e.g. heart value) into our study design for comparative analysis. More specifically, the cardiac fibroblast-like cell type, which would have represented an interesting comparison for further elucidation of shared phylogenetic relationships and specialized mechanisms for cellular plasticity and ECM regulation. The lack of inclusion of this cell type was due to two reasons: (1) we were limited in tissue procurement locations, and (2) discoveries of the genetic similarities between the VVF and cardiac development were previously unknown. Another caveat of this work is that our results were completed with culture fibroblasts from the tissue of interest (albeit at very low passages). Subsequently, gene expression from in vivo fibroblasts may not be entirely consistent with in vitro fibroblasts. Lastly, due to the inherent difficulty in accessing the larynx and acquisition of vocal fold tissue, we were unable to complete histological validation of our differential findings, however, recent work has demonstrated high sensitivity and reproducibility of RNA-seq, particularly in differential gene-expression analysis [106].

## Conclusions

The vocal folds are uniquely positioned at the crossroads of the unified airway with exposure to chronic insult resulting from environmental irritants [107] as well as excessive mechanical loads that warrant a distinct cellular phenotype capable of active remodeling during homeostasis and following wound injury [9–13]. Significant advances have been made in the recent years in understanding the pathophysiology of the stratified layers of the vocal fold, however major challenges continue to exist within the field of tissue engineering and developmental genetics. To our knowledge, this is the first investigation to sequence numerous human fibroblast cell types derived from various, albeit, shared developmental origins while using the VFF as a surrogate cell to evolve our understanding of its genotype as it relates to a mechanically stressed and differentiated cell type. Data was extrapolated in relation to biological processes for homeostatic maintenance of its unique structure. Upregulated VFF gene transcripts were associated with GO enrichment analyses which revealed several functional themes across various cell type comparisons related to transcription factors for signaling pathways regulating pluripotency of stem cells, as well as, ECM components of cell signaling, migration, proliferation, and differentiation potential. Human fibroblasts display a phenomenon of global topographic differentiation, which is maintained in isolation via in vitro assays. Epigenetic mechanical influences on vocal fold tissue may play a role in uniquely modelling and maintaining the local environmental cellular niche during homeostasis. VFF exhibited a distinctly specialized genotype related to their anatomic positional and developmental origins. Undoubtedly set forth during embryogenesis, the VFF genotype seems increasingly poised for cellular physiologic modifications for rapid and optimal response to local bioenvironmental fluctuations (e.g. inflammation) and various biomechanical demands.

## Methods

### Fibroblast isolation and culture

Primary normal human fibroblasts were derived from procurement of autopsy samples from healthy cadavers at University of Wisconsin-Madison, Department of Pathology and Laboratory Medicine in accordance and with approval from the Institutional Review Board (No: 2015–1482) at the University of Wisconsin-Madison. Additional human lung fibroblast cells from normal donors were kindly gifted from Dr. Carol Feghali Bostwick of the Medical University at South Carolina. Heterogeneous donors were included to serve as biological replicates rather than technical replicates. Primary cell lines assessed in this investigation represented fibroblasts along the unified airway which included; vocal fold (unilateral, anterior 1/3rd), trachea (superior to 3rd tracheal ring), lung, gingiva (upper), and palate (soft) as well as the inclusion of abdominal and scalp tissue samples for dermal site correlates. All tissue samples were procured from donors within 24 h of death and processed within 1 h of sample harvesting. Criteria for inclusion consisted of the following: (1) subjects were ≤ 89 years of age, and (2) were unaffected from any disease processes identified from medical records and visual inspection. For tissue explant and culture methodology, please refer to previously published work [5, 108, 109]. To allow for optimized cell proliferation and migration from original explants, medium was renewed every 2 to 3 days. Upon reaching subconfluence (e.g. 80–90% confluent), cells were trypsinized and passed to T75 flasks for downstream cell seeding. Passaging ratios between 1:2 and 1:4 were utilized for seeding of cells into 10 cm culture dishes at defined cell densities based upon manual counting of trypsinized cell suspensions using a hemocytometer and subsequent cell concentration calculations. For this experiment, fibroblast cells from passage 3 and 4 were utilized for RNA harvest.

### Fibroblast lineage confirmation utilizing endothelial, epithelial, and skeletal cell cultures

Human umbilical vein endothelial cells (HUVEC) (Lonza Walkersville, Inc.), human small airway epithelial cells (SAEC) (Lonza Walkersville, Inc., Walkersville, MD), and skeletal muscle cells (SkMC) (Lonza Walkersville, Inc.) were harvested from cryopreserved batches from our laboratory, plated and grown confluent with the following mediums: EGM-2 Bulletkit, EBM-2 plus SingleQuots® of growth supplements (CC-3162) (Lonza Walkersville, Inc.), SAGM Bulletkit, SABM plus SingleQuots® of growth supplements (CC-3118) (Lonza Walkersville, Inc.), and skeletal muscle cell supplemented growth medium (Cat No. 151–500) (Cell Applications, Inc.).

### Fluorescent immunocytochemistry

As previously published [5], a subtractive immunocytochemical methodology was utilized to resolve cell lineage by staining with von Willebrand factor (vWF), cytokeratin 19, and α-actinin antibodies for identification of endothelial, epithelial, and skeletal muscle cells, respectively. Fibroblasts were distinguished by the absence of staining for vWF, cytokeratin 19, and α-actinin. Staining was repeated in triplicate and in parallel for all cell lineages and cell types. SAEC, HUVEC, SkMC, and fibroblasts were seeded into separate 12-well plates on top of sterile glass slides at a density of 5 X 10^3^ cells per mL or 1 X 10^4^ cells per mL and grown in a 37 °C incubator with 5% CO_2_ to between 50 to 90% confluence. Cells were rinsed 1X with phosphate-buffered saline (PBS). SAEC, SkMC, and corresponding fibroblasts were fixed with 4% paraformaldehyde (PFA) for 10 min, while HUVEC cells were fixed with ice-cold methanol for 15 min and air dried. SAEC and fibroblasts were rinsed 3X with PBS and then covered for 10 min in 0.5% Triton X-100 buffer for permeabilization, while HUVEC and SkMC were treated with 1% NP-40 buffer. After which, cells were blocked in a solution of 1% bovine serum albumin (BSA), 10% goat serum for 30 min.

All cells were then incubated, either in a 37 °C humidified incubator for 2 h or overnight at 4 °C, with the primary antibody of interest in 1% BSA (DAKO Corporation, Carpentaria, CA). Primary antibodies included the following: mouse antihuman cytokeratin 19 (1:200; DAKO Corporation) against SAEC, rabbit antihuman vWF (1:200, DAKO Corporation) against HUVEC, and mouse antihuman α-actinin (Sacromeric) (1:200; Sigma-Aldrich, St Louis, MO) against SkMC. All fibroblast cell types were incubated with all three primary antibodies in parallel with positive controls. Negative controls were also performed in parallel with normal rabbit serum (DAKO Corporation) or normal mouse serum (DAKO Corporation) at an equal concentration as the primary antibody. Following primary staining, cells were rinsed 3X with wash buffer (0.1% Triton X-100) and incubated at 21 °C for 1 h with fluorescein isothiocyanate–conjugated secondary goat antimouse or goat antirabbit antibody (1:200; BioSource International, Inc., Camarillo, CA) for green fluorescence. Cells were then mounted with VECTASHIELD Mounting Medium with propidium iodine or DAPI (Bector Laboratories, Inc., Burlingame, CA) to counterstain DNA fluorescent red or blue to confirm the presence of live cells. An additional image file displays all primary and secondary antibodies used in detail [see Additional file 4]. Immunofluorescence images were captured on a Nikon Eclipse E600 fluorescent microscope (Nikon, Melville, NY) with a Pixera color camera (Pixera, Los Gatos, CA). Images were collected at 10X and 20X and merged with cellSens digital imaging software v1.9 (Olympus). All images were captured with consistent exposure settings.

### Isolation and purification of Total RNA

To obtain total RNA, cultured fibroblasts were trypsinized and harvested at a density no less than 1 X 10^6^. Cells were then neutralized with fresh media (described earlier) and centrifuged at 1000 rpm for 10 min to retrieve the cell pellet. Total RNA was purified from cell pellet samples using the RNeasy Mini Kit (Cat No. 74106) (QIAGEN) according to the manufacturer’s protocol. RNase-free DNase (QIAGEN) was treated on the column for 15 min to remove the minimum genomic DNA contamination. Quantification of all total RNA samples, initially, were performed using a NanoDrop 1000 spectrophotometer (Thermo Scientific), and integrity of samples were confirmed with the following three criteria for inclusion: (1) a concentration > 40 ng/ml, (2) an A_260_/A_280_ rating of 1.8 and 2.1, and (3) an A_260_/A_230_ ratio > 1.8.

### Construction and sequencing of directional libraries

Total RNA submitted to the University of Wisconsin-Madison Biotechnology Center was verified for purity and integrity via the NanoDrop One Spectrophotometer and Agilent 2100 BioAnalyzer, respectively. Samples that met the Illumina sample input guidelines were prepared according the TruSeq® Stranded mRNA Sample Preparation Guide (Rev. E) using the Illumina® TruSeq® Stranded mRNA Sample Preparation kits (Illumina Inc., San Diego, California, USA). For each library preparation, mRNA was purified from 1μg total RNA using poly-T oligo-attached magnetic beads. Subsequently, each poly-A enriched sample was fragmented using divalent cations under increased temperature. The fragmented RNA was synthesized into double-stranded cDNA using SuperScript II Reverse Transcriptase (Invitrogen, Carlsbad, California, USA) and random primers for first strand cDNA synthesis followed by second strand synthesis using DNA Polymerase I and RNAse H for removal of mRNA. Double-stranded cDNA was purified by paramagnetic beads (Agencourt AMPure XP beads, Beckman Coulter). The cDNA products were incubated with Klenow DNA Polymerase to add an ‘A’ base (Adenine) to the 3′ end of the blunt DNA fragments. DNA fragments were ligated to Illumina adapters, which have a single ‘T’ base (Thymine) overhang at their 3′ end. Adapter-ligated DNA products were purified by paramagnetic beads, successively amplified in a Linker Mediated PCR reaction (LM-PCR) for nine cycles using Phusion™ DNA Polymerase and Illumina’s PE genomic DNA primer set and then purified by paramagnetic beads. Quantity and quality of completed libraries were evaluated using a Qubit® dsDNA HS Assay Kit (Invitrogen, Carlsbad, California, USA), and an Agilent HS DNA or DNA1000 chip (Agilent Technologies, Inc., Santa Clara, CA, USA), respectively. Libraries were standardized to 2 nM. Cluster generation was executed using standard Cluster Kits (v4) and the Illumina cBot. Single end, 100 bp sequencing was completed using standard SBS chemistry (v4) on an Illumina HiSeq2500 sequencer. Images were analyzed using the standard Illumina Pipeline, version 1.8.2.

### Statistical analysis

Single end samples were sequenced using one lane; 88% of the reads on average were mapped back to the transcriptome yielding 29,155,924 as the average mapped reads and 33,073,817 as the average of total reads using the short-read aligner Bowtie (version 1.0.0) [110], followed by RSEM (version 1.2.7) to estimate gene expression [111]. All analyses were carried out in R (version 3.4.0; R Development Core Team, 2012), with specific software packages obtained from Bioconductor [112]. Data were normalized using Median by Ratio method in EBSeq (version 1.14.0) [113] and DESeq2 (version 1.16.1) was applied for identification of differentially expressed genes [114]. For each heatmap, the rows are transformed to z-scores. Specifically, expression in each row is scaled to have mean zero and standard deviation one. Hierarchical clustering and principle component analysis (PCA) were used to visualize the overall effect of group, age, and sex. Age-related effects were analyzed following the grouping of patients into three main categories: < 40, 40–69, and ≥ 70. *P*-values were adjusted by the Benjamini-Hochberg method to control False Discovery Rate (FDR) at 0.05.

### Enrichment analysis

Once exclusively differentially expressed genes were identified, we performed tests of enrichment using Gene Ontology (GO) annotations utilizing Enrichr (version 2017b) [115, 116] to investigate evidence of overrepresentation of common ontology terms for all cell type comparisons. Enrichr software filters GO cellular component and biologic process results by a “combined score” which represents the log of the *p*-value from the Fisher’s Exact test multiplied by the z-score of the deviation from the expected rank and with the ontology tree cut at a level four for generation of gene sets. KEGG pathway and WikiPathway analyses within the Enrichr software are computed by their respective p-value from the Fisher’s Exact test.

## Additional files

Additional file 1: Most highly expressed gene transcripts per anatomic site comparison. Top 10% identified significantly differentially expressed gene transcripts within each cluster for upregulated vocal fold condition versus trachea; top 2% for vocal fold versus lung; top 5% for vocal fold versus soft palate; and top 5% for vocal fold versus upper gingiva cell type comparisons. Fold change in DE and associated adjusted *P*-value are indicated. (TIF 544 kb)

Additional file 2: Differential gene expression pattern analysis for dermal comparisons identified by RNA sequencing. (A) Transcriptomic heatmap exhibiting clustering of 3352 genes differentially expressed between vocal fold versus scalp dermis. (B) Transcriptomic heatmap exhibiting clustering of 3471 genes differentially expressed between vocal fold versus abdomen dermis. Adjusted *P* < 0.05. Rainbow colored dendrogram panel represents clustering of genes, where closely related genes will be grouped together. Genes within a cluster are in a similar color and more correlated to each other than to genes outside that cluster. (TIF 853 kb)

Additional file 3: Gene specific heatmaps. (A) Human heart development comparing gene transcripts for vocal fold, lung, palate, and gingiva fibroblast cell types, as well as, (B) ECM disassembly comparing gene transcripts for vocal fold, palate, and gingiva fibroblast cell types. Upregulated gene transcripts were identified by WikiPathways analysis and GO biologic process analysis within Enrichr software. (TIF 241 kb)

Additional file 4: Primary and secondary antibodies. (TIF 286 kb)

## Abbreviations

BSA: Bovine serum albumin
cAMP: Cyclic adenosine monophosphate
cDNA: Complementary deoxyribonucleic acid
CPF: Cardiopharyngeal field
DAPI: 4′,6-diamidino-2-phenylindole
DE: Differentially expressed
DNA: Deoxyribonucleic acid
ECM: Extracellular matrix
ESMs: Esophagus striated muscles
ET-1: Endothelin-1
FGF: Fibroblast growth factor
FHF: First-heart-field
GO: Gene ontology
HA: Hyaluronic acid
HAS: Hyaluronic acid synthase
HUVEC: Human umbilical vein endothelial cells
IL-8: Interleukin-8
KEGG: Kyoto Encyclopedia of Genes and Genomes
LP: Lamina propria
MAC: Membrane attack complex
MCP-1: Monocyte chemoattractant protein-1
MMPs: Matrix metalloproteinases
mRNA: messenger ribonucleic acid
MSC: Mesenchymal stem cell
PBS: Phosphate-buffered saline
PCA: Principle component analysis
PFA: Paraformaldehyde
Rap1: Ras-related protein 1
RNA: Ribonucleic acid
SAEC: Small airway epithelial cells
SHF: Second-heart-field
SkMC: Skeletal muscle cells
SLRP: Small leucine-rich proteoglycan
TGFb: Transforming growth factor beta
TLR: Toll-like receptors
VF: Vocal folds
VFF: Vocal fold fibroblast
vWF: Von Willebrand

## References

[1] Mammoto A, Mammoto T, Ingber DE (2012). Mechanosensitive mechanisms in transcriptional regulation. J Cell Sci.

[2] Kessler D, Dethlefsen S, Haase I, Plomann M, Hirche F, Krieg T, Eckes B (2001). Fibroblasts in mechanically stressed collagen lattices assume a “synthetic” phenotype. J Biol Chem.

[3] Eastwood M, McGrouther DA, Brown RA (1998). Fibroblast responses to mechanical forces. Proc Inst Mech Eng H.

[4] Chiquet M, Gelman L, Lutz R, Maier S (2009). From mechanotransduction to extracellular matrix gene expression in fibroblasts. Biochim Biophys Acta.

[5] Thibeault SL, Li W, Bartley S (2008). A method for identification of vocal fold lamina propria fibroblasts in culture. Otolaryngol Head Neck Surg.

[6] Rinn JL, Bondre C, Gladstone HB, Brown PO, Chang HY (2006). Anatomic demarcation by positional variation in fibroblast gene expression programs. PLoS Genet.

[7] Higuchi Y, Kojima M, Ishii G, Aoyagi K, Sasaki H, Ochiai A (2015). Gastrointestinal fibroblasts have specialized, diverse transcriptional phenotypes: a comprehensive gene expression analysis of human fibroblasts. PLoS One.

[8] Chang HY, Chi JT, Dudoit S, Bondre C, van de Rijn M, Botstein D, Brown PO (2002). Diversity, topographic differentiation, and positional memory in human fibroblasts. Proc Natl Acad Sci U S A.

[9] Wang JH, Thampatty BP, Lin JS, Im HJ (2007). Mechanoregulation of gene expression in fibroblasts. Gene.

[10] Titze IR, Hitchcock RW, Broadhead K, Webb K, Li W, Gray SD, Tresco PA (2004). Design and validation of a bioreactor for engineering vocal fold tissues under combined tensile and vibrational stresses. J Biomech.

[11] Gaston J, Quinchia Rios B, Bartlett R, Berchtold C, Thibeault SL (2012). The response of vocal fold fibroblasts and mesenchymal stromal cells to vibration. PLoS One.

[12] Chiquet M, Reneda AS, Huber F, Fluck M (2003). How do fibroblasts translate mechanical signals into changes in extracellular matrix production?. Matrix Biol.

[13] Bartlett RS, Gaston JD, Yen TY, Ye S, Kendziorski C, Thibeault SL (2015). Biomechanical screening of cell therapies for vocal fold scar. Tissue Eng Part A.

[14] McAnulty RJ (2007). Fibroblasts and myofibroblasts: their source, function and role in disease. Int J Biochem Cell B.

[15] Ding H, Gray SD (2001). Senescent expression of genes coding collagens, collagen-degrading metalloproteinases, and tissue inhibitors of metalloproteinases in rat vocal folds: comparison with skin and lungs. J Gerontol a-Biol.

[16] Xu QC, Kuang RX, Wei SQ, Kang Q, Wang JJ, Wang ZG (2017). Analysis of mechanical behavior of dermal fibroblasts obtained from various anatomical sites in humans. Ann Plast Surg.

[17] Kuang R, Wang Z, Xu Q, Liu S, Zhang W (2015). Influence of mechanical stimulation on human dermal fibroblasts derived from different body sites. Int J Clin Exp Med.

[18] Titze IR (1989). On the relation between subglottal pressure and fundamental-frequency in phonation. J Acoust Soc Am.

[19] Titze IR, Svec JG, Popolo PS (2003). Vocal dose measures: quantifying accumulated vibration exposure in vocal fold tissues. J Speech Lang Hear Res.

[20] Gray SD (2000). Cellular physiology of the vocal folds. Otolaryngol Clin N Am.

[21] Mayne BT, Bianco-Miotto T, Buckberry S, Breen J, Clifton V, Shoubridge C, Roberts CT (2016). Large scale gene expression meta-analysis reveals tissue-specific, sex-biased gene expression in humans. Front Genet.

[22] Issa JP (2003). Age-related epigenetic changes and the immune system. Clin Immunol.

[23] Issa JP (2002). Epigenetic variation and human disease. J Nutr.

[24] Hekimi S, Guarente L (2003). Genetics and the specificity of the aging process. Science.

[25] Chen X, Thibeault SL (2008). Characteristics of age-related changes in cultured human vocal fold fibroblasts. Laryngoscope.

[26] Kim YJ, Lim H, Li Z, Oh Y, Kovlyagina I, Choi IY, Dong X, Lee G (2014). Generation of multipotent induced neural crest by direct reprogramming of human postnatal fibroblasts with a single transcription factor. Cell Stem Cell.

[27] Lighthouse JK, Small EM (2016). Transcriptional control of cardiac fibroblast plasticity. J Mol Cell Cardiol.

[28] Chen WJ, Ho CC, Chang YL, Chen HY, Lin CA, Ling TY, Yu SL, Yuan SS, Chen YJ, Lin CY (2014). Cancer-associated fibroblasts regulate the plasticity of lung cancer stemness via paracrine signalling. Nat Commun.

[29] Branco A, Bartley SM, King SN, Jette ME, Thibeault SL (2016). Vocal fold myofibroblast profile of scarring. Laryngoscope.

[30] Mahankali M, Peng HJ, Cox D, Gomez-Cambronero J (2011). The mechanism of cell membrane ruffling relies on a phospholipase D2 (PLD2), Grb2 and Rac2 association. Cell Signal.

[31] Ridley AJ, Paterson HF, Johnston CL, Diekmann D, Hall A (1992). The small GTP-binding protein rac regulates growth factor-induced membrane ruffling. Cell.

[32] Suen PW, Ilic D, Caveggion E, Berton G, Damsky CH, Lowell CA (1999). Impaired integrin-mediated signal transduction, altered cytoskeletal structure and reduced motility in Hck/Fgr deficient macrophages. J Cell Sci.

[33] Weir MC, Shu ST, Patel RK, Hellwig S, Chen L, Tan L, Gray NS, Smithgall TE (2018). Selective inhibition of the myeloid Src-family kinase Fgr potently suppresses AML cell growth in vitro and in vivo. ACS Chem Biol.

[34] Shah S, Brock EJ, Ji K, Mattingly RR (2018). Ras and Rap1: a tale of two GTPases. Semin Cancer Biol.

[35] Yang Y, Zhang J, Yan Y, Cai H, Li M, Sun K, Wang JZ, Liu X, Wang JS, Duan XY (2017). Low expression of Rap1GAP is associated with epithelial-mesenchymal transition (EMT) and poor prognosis in gastric cancer. Oncotarget.

[36] Tsygankova OM, Wang H, Meinkoth JL (2013). Tumor cell migration and invasion are enhanced by depletion of Rap1 GTPase-activating protein (Rap1GAP). J Biol Chem.

[37] Zuo H, Gandhi M, Edreira MM, Hochbaum D, Nimgaonkar VL, Zhang P, DiPaola J, Evdokimova V, Altschuler DL, Nikiforov YE (2010). Downregulation of Rap1GAP through epigenetic silencing and loss of heterozygosity promotes invasion and progression of thyroid tumors. Cancer Res.

[38] Zheng H, Gao L, Feng Y, Yuan L, Zhao H, Cornelius LA (2009). Down-regulation of Rap1GAP via promoter hypermethylation promotes melanoma cell proliferation, survival, and migration. Cancer Res.

[39] Zhang Z, Mitra RS, Henson BS, Datta NS, McCauley LK, Kumar P, Lee JS, Carey TE, D'Silva NJ (2006). Rap1GAP inhibits tumor growth in oropharyngeal squamous cell carcinoma. Am J Pathol.

[40] Zhang L, Chenwei L, Mahmood R, van Golen K, Greenson J, Li G, D'Silva NJ, Li X, Burant CF, Logsdon CD (2006). Identification of a putative tumor suppressor gene Rap1GAP in pancreatic cancer. Cancer Res.

[41] Vuchak LA, Tsygankova OM, Meinkoth JL (2011). Rap1GAP impairs cell-matrix adhesion in the absence of effects on cell-cell adhesion. Cell Adhes Migr.

[42] Adams TE, Epa VC, Garrett TPJ, Ward CW (2000). Structure and function of the type 1 insulin-like growth factor receptor. Cell Mol Life Sci.

[43] Guvakova MA, Lee WSY, Furstenau DK, Prabakaran I, Li DC, Hung R, Kushnir N (2014). The small GTPase Rap1 promotes cell movement rather than stabilizes adhesion in epithelial cells responding to insulin-like growth factor I. Biochem J.

[44] Zhou H, Felsen D, Sandulache VC, Amin MR, Kraus DH, Branski RC (2011). Prostaglandin (PG) E2 exhibits antifibrotic activity in vocal fold fibroblasts. Laryngoscope.

[45] Wilgus TA, Vodovotz Y, Vittadini E, Clubbs EA, Oberyszyn TM (2003). Reduction of scar formation in full-thickness wounds with topical celecoxib treatment. Wound Repair Regen.

[46] Togo S, Holz O, Liu X, Sugiura H, Kamio K, Wang X, Kawasaki S, Ahn Y, Fredriksson K, Skold CM (2008). Lung fibroblast repair functions in patients with chronic obstructive pulmonary disease are altered by multiple mechanisms. Am J Respir Crit Care Med.

[47] Liu X, Nelson A, Wang X, Farid M, Gunji Y, Ikari J, Iwasawa S, Basma H, Feghali-Bostwick C, Rennard SI (2014). Vitamin D modulates prostaglandin E2 synthesis and degradation in human lung fibroblasts. Am J Respir Cell Mol Biol.

[48] Keerthisingam CB, Jenkins RG, Harrison NK, Hernandez-Rodriguez NA, Booth H, Laurent GJ, Hart SL, Foster ML, McAnulty RJ (2001). Cyclooxygenase-2 deficiency results in a loss of the anti-proliferative response to transforming growth factor-beta in human fibrotic lung fibroblasts and promotes bleomycin-induced pulmonary fibrosis in mice. Am J Pathol.

[49] Branski RC, Zhou H, Sandulache VC, Chen J, Felsen D, Kraus DH (2010). Cyclooxygenase-2 signaling in vocal fold fibroblasts. Laryngoscope.

[50] Necas J, Bartosikova L, Brauner P, Kolar J (2008). Hyaluronic acid (hyaluronan): a review. Vet Med-Czech.

[51] Ohkawara Y, Tamura G, Iwasaki T, Tanaka A, Kikuchi T, Shirato K (2000). Activation and transforming growth factor-beta production in eosinophils by hyaluronan. Am J Respir Cell Mol.

[52] McKee CM, Lowenstein CJ, Horton MR, Wu J, Bao C, Chin BY, Choi AM, Noble PW (1997). Hyaluronan fragments induce nitric-oxide synthase in murine macrophages through a nuclear factor κB-dependent mechanism. J Biol Chem.

[53] Lees VC, Fan TPD, West DC (1995). Angiogenesis in a delayed revascularization model is accelerated by Angiogenic oligosaccharides of Hyaluronan. Lab Investig.

[54] Hodge-Dufour J, Noble PW, Horton MR, Bao C, Wysoka M, Burdick MD, Strieter RM, Trinchieri G, Puré E (1997). Induction of IL-12 and chemokines by hyaluronan requires adhesion-dependent priming of resident but not elicited macrophages. J Immunol.

[55] Ward PD, Thibeault SL, Gray SD (2002). Hyaluronic acid: its role in voice. J Voice.

[56] Thibeault SL, Gray SD, Bless DM, Chan RW, Ford CN (2002). Histologic and rheologic characterization of vocal fold scarring. J Voice.

[57] Chan RW, Gray SD, Titze IR (2001). The importance of hyaluronic acid in vocal fold biomechanics. Otolaryngol Head Neck Surg.

[58] Kennedy CI, Diegelmann RF, Haynes JH, Yager DR (2000). Proinflammatory cytokines differentially regulate hyaluronan synthase isoforms in fetal and adult fibroblasts. J Pediatr Surg.

[59] Longaker MT, Chiu ES, Adzick NS, Stern M, Harrison MR, Stern R (1991). Studies in fetal wound healing. V. a prolonged presence of hyaluronic acid characterizes fetal wound fluid. Ann Surg.

[60] Tammi R, Pasonen-Seppanen S, Kolehmainen E, Tammi M (2005). Hyaluronan synthase induction and hyaluronan accumulation in mouse epidermis following skin injury. J Invest Dermatol.

[61] Podack ER (1984). Molecular composition of the tubular structure of the membrane attack complex of complement. J Biol Chem.

[62] Serna M, Giles JL, Morgan BP, Bubeck D (2016). Structural basis of complement membrane attack complex formation. Nat Commun.

[63] Mak BC, McConkey F, Feng N, O'Reilly K, Kasprzyk PG, Rubinstein D, Hahn SE, Pereira DS, Findlay H, Young DS (2007). AR36A36.11.1, a monoclonal antibody targeting CD59, enhances complement activity and exhibits potent in vivo efficacy in multiple human cancer models. Mol Cancer Ther.

[64] Emin M, Wang G, Castagna F, Rodriguez-Lopez J, Wahab R, Wang J, Adams T, Wei Y, Jelic S (2016). Increased internalization of complement inhibitor CD59 may contribute to endothelial inflammation in obstructive sleep apnea. Sci Transl Med.

[65] Wyatt A, Yerbury J, Dabbs R, Wilson M (2009). The chaperone action of Clusterin and its putative role in quality control of extracellular protein folding. Adv Cancer Res.

[66] Shannan B, Seifert M, Boothman DA, Tilgen W, Reichrath J (2006). Clusterin and DNA repair: a new function in cancer for a key player in apoptosis and cell cycle control. J Mol Histol.

[67] Leskov KS, Klokov DY, Li J, Kinsella TJ, Boothman DA (2003). Synthesis and functional analyses of nuclear clusterin, a cell death protein. J Biol Chem.

[68] Urbich C, Fritzenwanger M, Zeiher AM, Dimmeler S (2000). Laminar shear stress upregulates the complement-inhibitory protein clusterin: a novel potent defense mechanism against complement-induced endothelial cell activation. Circulation.

[69] Lane TF, Sage EH (1994). The biology of Sparc, a protein that modulates cell-matrix interactions. FASEB J.

[70] Brekken RA, Sage EH (2000). SPARC, a matricellular protein: at the crossroads of cell–matrix. Matrix Biol.

[71] Duflo SM, Thibeault SL, Li W, Smith ME, Schade G, Hess MM (2006). Differential gene expression profiling of vocal fold polyps and Reinke's edema by complementary DNA microarray. Ann Otol Rhinol Laryngol.

[72] Tai IT, Tang MJ (2008). SPARC in cancer biology: its role in cancer progression and potential for therapy. Drug Resist Updat.

[73] Dameron KM, Volpert OV, Tainsky MA, Bouck N (1994). Control of angiogenesis in fibroblasts by P53 regulation of Thrombospondin-1. Science.

[74] Agah A, Kyriakides TR, Lawler J, Bornstein P (2002). The lack of thrombospondin-1 (TSP1) dictates the course of wound healing in double-TSP1/TSP2-null mice. Am J Pathol.

[75] Troen BR (2006). The regulation of cathepsin K gene expression. Ann N Y Acad Sci.

[76] Saika S, Shiraishi A, Saika S, Liu CY, Funderburgh JL, Kao CWC, Converse RL, Kao WWY (2000). Role of lumican in the corneal epithelium during wound healing. J Biol Chem.

[77] Iozzo RV (1999). The biology of the small leucine-rich proteoglycans - functional network of interactive proteins. J Biol Chem.

[78] Chen S, Birk DE (2013). The regulatory roles of small leucine-rich proteoglycans in extracellular matrix assembly. FEBS J.

[79] Gray SD, Titze IR, Chan R, Hammond TH (1999). Vocal fold proteoglycans and their influence on biomechanics. Laryngoscope.

[80] Westergren-Thorsson G, Hernnäs J, Särnstrand B, Oldberg A, Heinegård D, Malmström A (1993). Altered expression of small proteoglycans, collagen, and transforming growth factor-beta 1 in developing bleomycin-induced pulmonary fibrosis in rats. J Clin Invest.

[81] Wrana JL, Overall CM, Sodek J (1991). Regulation of the expression of a secreted acidic protein-rich in cysteine (Sparc) in human fibroblasts by transforming growth-Factor-Beta - comparison of transcriptional and posttranscriptional control with fibronectin and type-I collagen. Eur J Biochem.

[82] Yang J, Savvatis K, Kang JS, Fan PD, Zhong HY, Schwartz K, Barry V, Mikels-Vigdal A, Karpinski S, Kornyeyev D (2016). Targeting LOXL2 for cardiac interstitial fibrosis and heart failure treatment. Nat Commun.

[83] Doherty JM, Geske MJ, Stappenbeck TS, Mills JC (2008). Diverse adult stem cells share specific higher-order patterns of gene expression. Stem Cells.

[84] Tzahor E (2015). Head muscle development. Results Probl Cell Differ.

[85] Wachtler F, Jacob M. Origin and development of the cranial skeletal muscles. Bibl Anat. 1986;(29):24–46. 3729921

[86] Romer AS (1950). The vertebrate body.

[87] Harel I, Maezawa Y, Avraham R, Rinon A, Ma HY, Cross JW, Leviatan N, Hegesh J, Roy A, Jacob-Hirsch J (2012). Pharyngeal mesoderm regulatory network controls cardiac and head muscle morphogenesis. Proc Natl Acad Sci U S A.

[88] Diogo R, Kelly RG, Christiaen L, Levine M, Ziermann JM, Molnar JL, Noden DM, Tzahor E (2015). A new heart for a new head in vertebrate cardiopharyngeal evolution. Nature.

[89] Waldo KL, Kumiski DH, Wallis KT, Stadt HA, Hutson MR, Platt DH, Kirby ML (2001). Conotruncal myocardium arises from a secondary heart field. Development.

[90] Mjaatvedt CH, Nakaoka T, Moreno-Rodriguez R, Norris RA, Kern MJ, Eisenberg CA, Turner D, Markwald RR (2001). The outflow tract of the heart is recruited from a novel heart-forming field. Dev Biol.

[91] Kelly RG, Brown NA, Buckingham ME (2001). The arterial pole of the mouse heart forms from Fgf10-expressing cells in pharyngeal mesoderm. Dev Cell.

[92] Harel I, Nathan E, Tirosh-Finkel L, Zigdon H, Guimaraes-Camboa N, Evans SM, Tzahor E (2009). Distinct origins and genetic programs of head muscle satellite cells. Dev Cell.

[93] Watanabe Y, Zaffran S, Kuroiwa A, Higuchi H, Ogura T, Harvey RP, Kelly RG, Buckingham M (2012). Fibroblast growth factor 10 gene regulation in the second heart field by Tbx1, Nkx2-5, and Islet1 reveals a genetic switch for down-regulation in the myocardium. Proc Natl Acad Sci USA.

[94] Prall OWJ, Menon MK, Solloway MJ, Watanabe Y, Zaffran S, Bajolle F, Biben C, McBride JJ, Robertson BR, Chaulet H (2007). An Nkx2-5/Bmp2/Smad1 negative feedback loop controls heart progenitor specification and proliferation. Cell.

[95] Dodou E, Verzi MP, Anderson JR, Xu SM, Black BL (2004). Mef2c is a direct transcriptional target of ISL1 and GATA factors in the anterior heart field during mouse embryonic development. Development.

[96] Srivastava D (2006). Making or breaking the heart: from lineage determination to morphogenesis. Cell.

[97] Yanagisawa H, Clouthier DE, Richardson JA, Charite J, Olson EN (2003). Targeted deletion of a branchial arch-specific enhancer reveals a role of dHAND in craniofacial development. Development.

[98] Osterwalder M, Speziale D, Shoukry M, Mohan R, Ivanek R, Kohler M, Beisel C, Wen XH, Scales SJ, Christoffels VM (2014). HAND2 targets define a network of transcriptional regulators that compartmentalize the early limb bud mesenchyme. Dev Cell.

[99] Lints TJ, Parsons LM, Hartley L, Lyons I, Harvey RP (1993). Nkx-2.5 - a novel murine Homeobox gene expressed in early heart progenitor cells and their myogenic descendants (Vol 119, Pg 419, 1993). Development.

[100] Hoffman TL, Javier AL, Campeau SA, Knight RD, Schilling TF (2007). Tfap2 transcription factors in zebrafish neural crest development and ectodermal evolution. J Exp Zool Part B.

[101] Zhang X, Bao L, Yang L, Wu QF, Li S (2012). Roles of intracellular fibroblast growth factors in neural development and functions. Sci China Life Sci.

[102] de Croze N, Maczkowiak F, Monsoro-Burq AH (2011). Reiterative AP2a activity controls sequential steps in the neural crest gene regulatory network. P Natl Acad Sci USA.

[103] Yamada M, Revelli JP, Eichele G, Barron M, Schwartz RJ (2000). Expression of chick Tbx-2, Tbx-3, and Tbx-5 genes during early heart development: evidence for BMP2 induction of Tbx2. Dev Biol.

[104] Douglas NC, Papaioannou VE (2013). The T-box transcription factors TBX2 and TBX3 in mammary gland development and breast Cancer. J Mammary Gland Biol.

[105] Merscher S, Funke B, Epstein JA, Heyer J, Puech A, Lu MM, Xavier RJ, Demay MB, Russell RG, Factor S (2001). TBX1 is responsible for cardiovascular defects in Velo-cardio-facial/DiGeorge syndrome. Cell.

[106] Consortium SM-I (2014). A comprehensive assessment of RNA-seq accuracy, reproducibility and information content by the sequencing quality control consortium. Nat Biotechnol.

[107] Thibeault SL, Rees L, Pazmany L, Birchall MA (2009). At the crossroads: mucosal immunology of the larynx. Mucosal Immunol.

[108] Ylitalo R, Baugh A, Li W, Thibeault S (2004). Effect of acid and pepsin on gene expression in laryngeal fibroblasts. Ann Otol Rhinol Laryngol.

[109] Thibeault SL, Smith ME, Peterson K, Ylitalo-Moller R (2007). Gene expression changes of inflammatory mediators in posterior laryngitis due to laryngopharyngeal reflux and evolution with PPI treatment: a preliminary study. Laryngoscope.

[110] Langmead B, Trapnell C, Pop M, Salzberg SL (2009). Ultrafast and memory-efficient alignment of short DNA sequences to the human genome. Genome Biol.

[111] Li B, Dewey CN (2011). RSEM: accurate transcript quantification from RNA-Seq data with or without a reference genome. Bmc Bioinformatics.

[112] Gentleman RC, Carey VJ, Bates DM, Bolstad B, Dettling M, Dudoit S, Ellis B, Gautier L, Ge Y, Gentry J (2004). Bioconductor: open software development for computational biology and bioinformatics. Genome Biol.

[113] Leng N, Dawson JA, Thomson JA, Ruotti V, Rissman AI, Smits BM, Haag JD, Gould MN, Stewart RM, Kendziorski C (2013). EBSeq: an empirical Bayes hierarchical model for inference in RNA-seq experiments. Bioinformatics.

[114] Love MI, Huber W, Anders S (2014). Moderated estimation of fold change and dispersion for RNA-seq data with DESeq2. Genome Biol.

[115] Kuleshov MV, Jones MR, Rouillard AD, Fernandez NF, Duan Q, Wang Z, Koplev S, Jenkins SL, Jagodnik KM, Lachmann A (2016). Enrichr: a comprehensive gene set enrichment analysis web server 2016 update. Nucleic Acids Res.

[116] Chen EY, Tan CM, Kou Y, Duan Q, Wang Z, Meirelles GV, Clark NR, Ma'ayan A (2013). Enrichr: interactive and collaborative HTML5 gene list enrichment analysis tool. BMC Bioinformatics.

