# Supplementary figures and images for: Tissue specific human fibroblast differential expression based on RNAsequencing analysis
Source: BMC Genomics. 2019 Apr 23;20:308. doi: 10.1186/s12864-019-5682-5 (PMC6480701; doi:10.1186/s12864-019-5682-5)

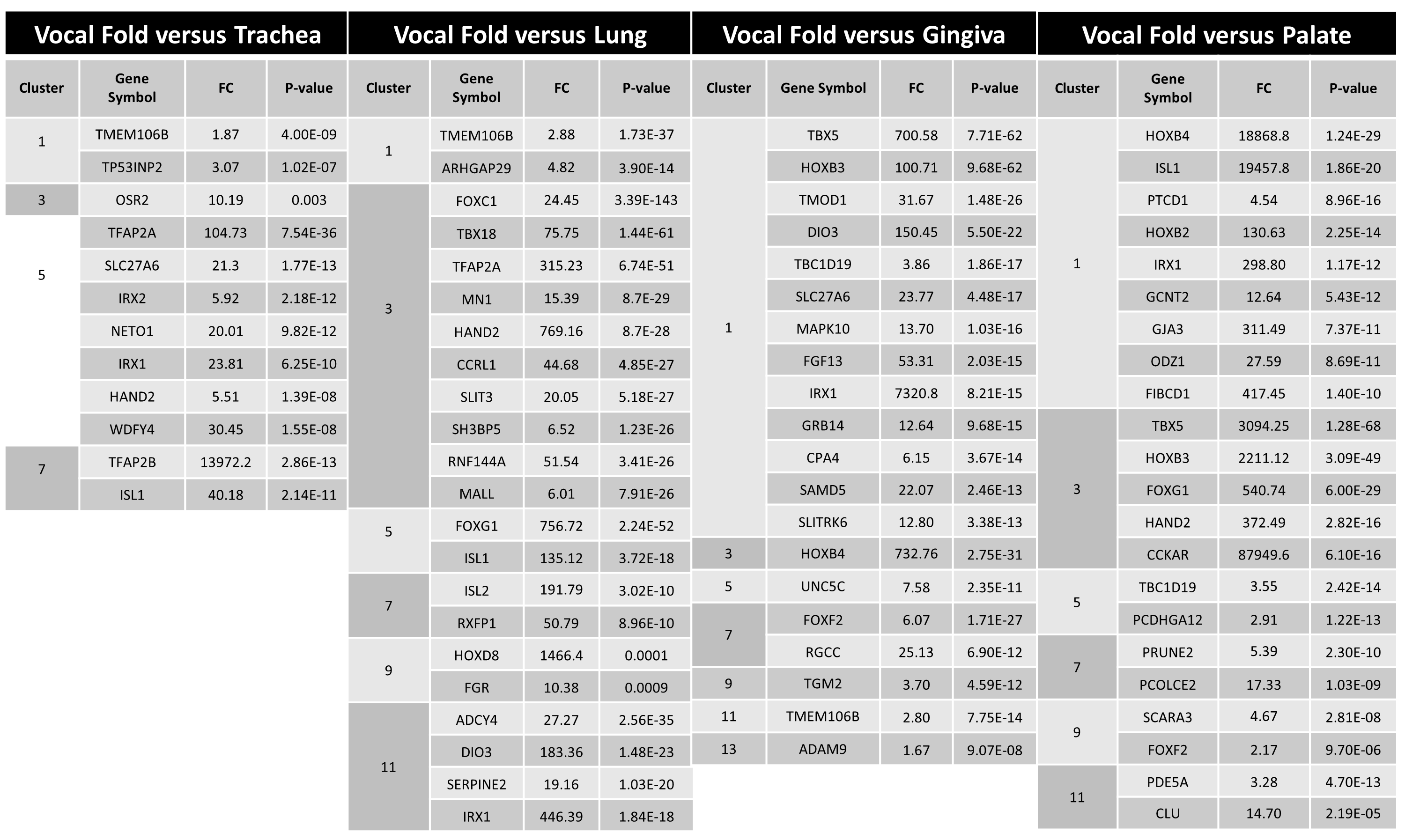

Supplement: Supplementary file 1 — Most highly expressed gene transcripts per anatomic site comparison. Top 10% identified significantly differentially expressed gene transcripts within each cluster for upregulated vocal fold condition versus trachea; top 2% for vocal fold versus lung; top 5% for vocal fold versus soft palate; and top 5% for vocal fold versus upper gingiva cell type comparisons. Fold change in DE and associated adjusted P-value are indicated. (TIF 544 kb) [file 12864_2019_5682_MOESM1_ESM.tif]

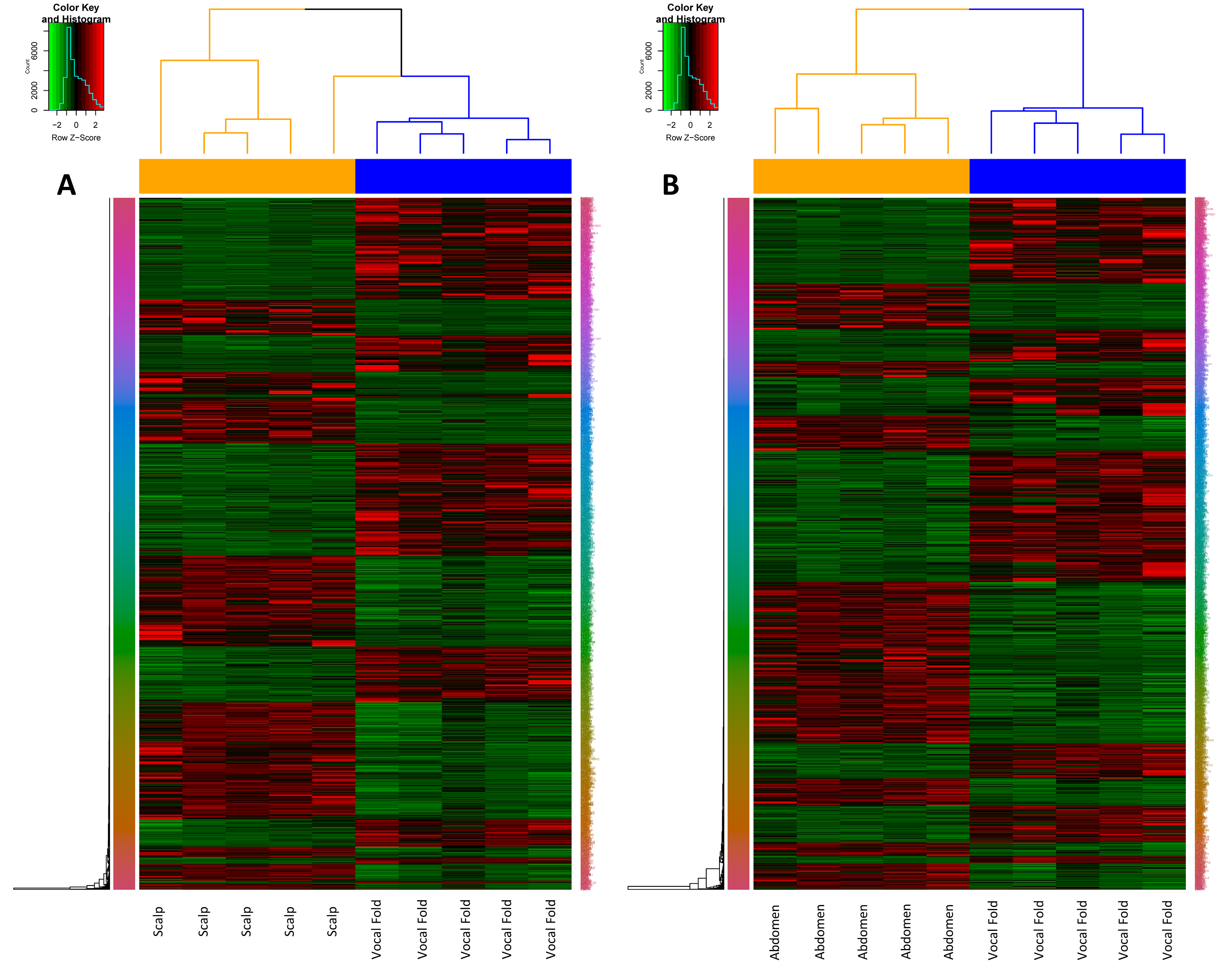

Supplement: Supplementary file 2 — Differential gene expression pattern analysis for dermal comparisons identified by RNA sequencing. (A) Transcriptomic heatmap exhibiting clustering of 3352 genes differentially expressed between vocal fold versus scalp dermis. (B) Transcriptomic heatmap exhibiting clustering of 3471 genes differentially expressed between vocal fold versus abdomen dermis. Adjusted P < 0.05. Rainbow colored dendrogram panel represents clustering of genes, where closely related genes will be grouped together. Genes within a cluster are in a similar color and more correlated to each other than to genes outside that cluster. (TIF 853 kb) [file 12864_2019_5682_MOESM2_ESM.tif]

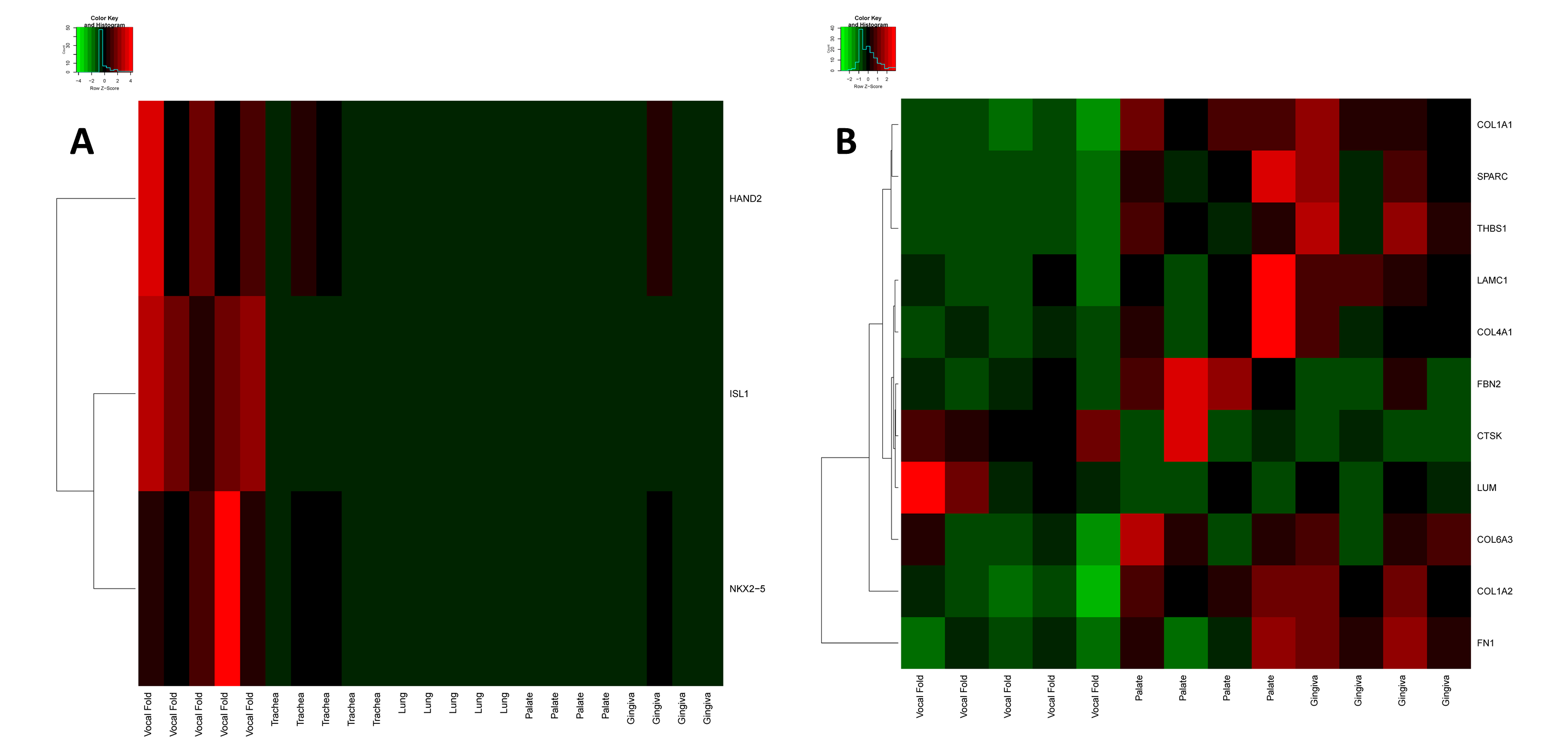

Supplement: Supplementary file 3 — Gene specific heatmaps. (A) Human heart development comparing gene transcripts for vocal fold, lung, palate, and gingiva fibroblast cell types, as well as, (B) ECM disassembly comparing gene transcripts for vocal fold, palate, and gingiva fibroblast cell types. Upregulated gene transcripts were identified by WikiPathways analysis and GO biologic process analysis within Enrichr software. (TIF 241 kb) [file 12864_2019_5682_MOESM3_ESM.tif]

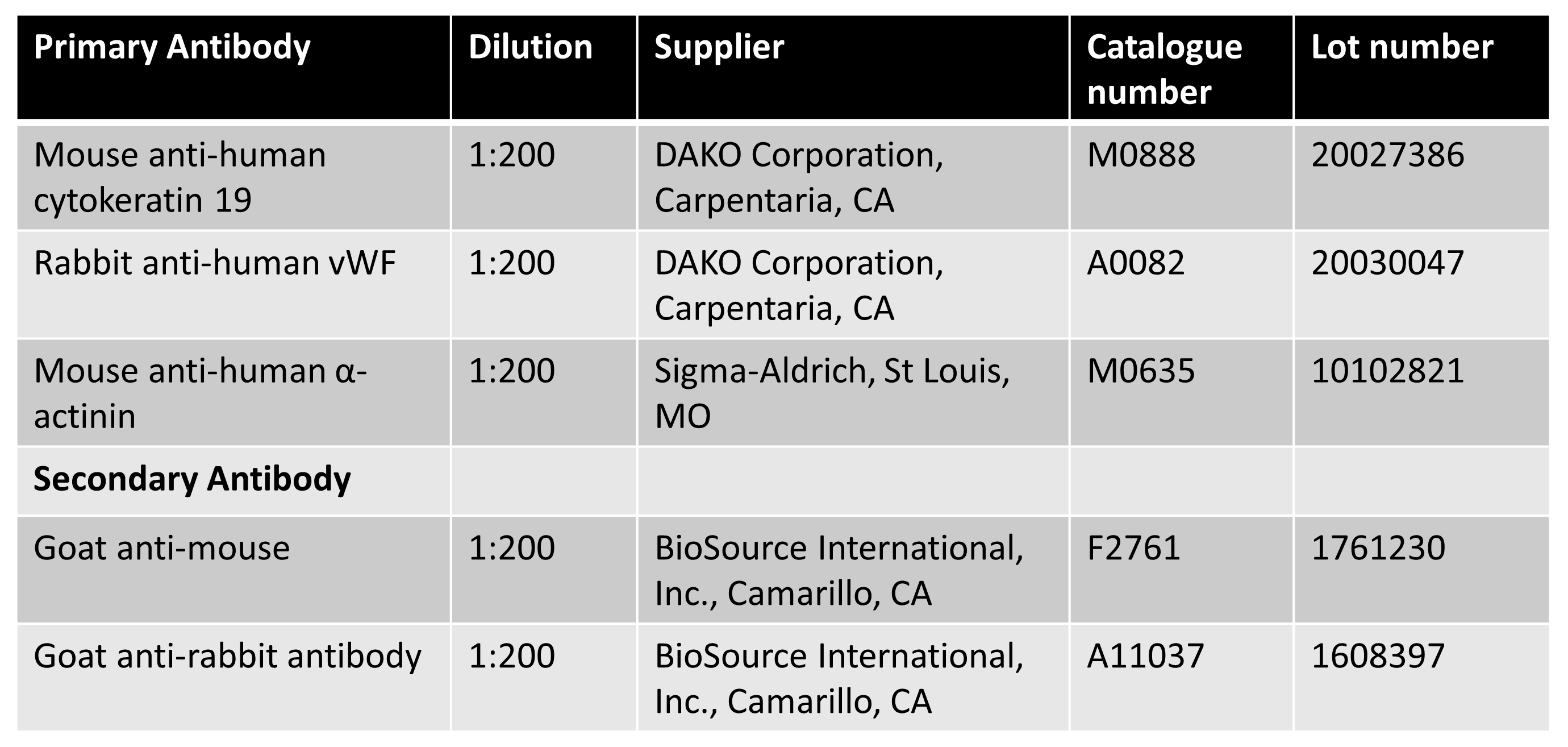

Supplement: Supplementary file 4 — Primary and secondary antibodies. (TIF 286 kb) [file 12864_2019_5682_MOESM4_ESM.tif]
